# Integrated multi-omics analysis deciphers the molecular mechanisms underlying the characteristic volatile flavor of Meishan pork

**DOI:** 10.1186/s40104-026-01515-7

**Published:** 2026-11-04

**Authors:** Huangbing Sun, Zhongyu Yang, Haoyang Wang, Pengju Du, Wangjun Wu, Guoqiang Fan, Xiaojing Yang

**Affiliations:** 1 MOE Joint International Research Laboratory of Animal Health & Food Safety, Nanjing Agricultural University, Nanjing, 210095 China; 2 Key Laboratory of Animal Physiology & Biochemistry, Ministry of Agriculture and Rural Affairs, Nanjing Agricultural University, Nanjing, 210095 China; 3 Department of Animal Genetics, Breeding and Reproduction, College of Animal Science and Technology, Nanjing Agricultural University, Nanjing, 210095 China

**Keywords:** ALOX5, Lipid oxidation, Meishan pig, Multi-omics, Volatile flavor compounds

## Abstract

**Background:**

The Meishan pig is a celebrated indigenous Chinese swine breed renowned for its distinctive flavor profile. However, the specific signature flavor compounds and the molecular mechanisms underlying their formation remain largely uncharacterized. This study aimed to comprehensively profile the volatile flavor compounds across five pig breeds and decipher the molecular basis of the characteristic flavor of Meishan pork.

**Results:**

Comparative analysis combining electronic nose, headspace solid-phase microextraction coupled with gas chromatography-mass spectrometry (HS-SPME-GC-MS), and relative odor activity value calculations revealed that Meishan pork contained significantly higher levels of four key aroma compounds: hexanal, nonanal, 2-pentylfuran, and (E,E)-2,4-nonadienal. Integrated metabolomic and transcriptomic analyses identified oleic acid and α-linolenic acid as critical precursors and highlighted the high expression of the arachidonate 5-lipoxygenase gene (*ALOX5*) in Meishan pigs. Heating experiments confirmed that these fatty acids yield characteristic flavor compounds upon thermal oxidation. Furthermore, functional studies demonstrated that *ALOX5* catalyzes the conversion of these precursors into flavor aldehydes.

**Conclusions:**

The distinctive flavor of Meishan pork is associated with a dual mechanism: thermal oxidation of abundant precursors during cooking, and enhanced enzymatic oxidation prior to cooking resulting from constitutively high *ALOX5* expression. By deciphering the flavor formation mechanism from metabolic precursors to enzyme-catalyzed conversion, this investigation provides a framework for elucidating meat flavor chemistry and offers potential targets for breeding strategies aimed at improving pork flavor.

**Supplementary Information:**

The online version contains supplementary material available at https://doi.org/10.1186/s40104-026-01515-7.

## Introduction

China dominates global pork production and consumption, constituting approximately half of worldwide output with annual per capita consumption exceeding 30 kg [1, 2]. Consumer preference and market value for pork are fundamentally determined by its quality, with the profile of volatile flavor compounds (VFCs) being a primary driver of sensory appeal and product differentiation. China holds a rich genetic reservoir of pig breeds, accounting for about 23.5% of the global diversity [3]. Chinese indigenous pig breeds are renowned for their superior meat quality and distinctive sensory attributes. Among them, Meishan pigs stand out as a representative breed, not only for their remarkable traits of roughage tolerance and high prolificacy, but also for their pork flavor, which is highly favored by consumers [4]. These attributes make them a prized genetic resource frequently utilized in breeding programs worldwide [5, 6]. Several indigenous breeds have been associated with specific flavor compounds, with reports of dodecanal, (E,E)-2,4-decadienal, 2,3‑octanedione, 1‑octen‑3‑ol, and hexanal distinguishing Laiwu pigs and Beijing Black pigs from DLY pigs [7], and higher abundances of 4‑pentylbenzaldehyde, 4‑heptenal, and heptanal found in Tibetan pork [8]. Despite these advances, the scientific understanding of key pork flavor profiles remains incomplete. Specifically for the prized Meishan pork, the signature flavor compounds that define its unique character have yet to be clearly deciphered.

Meat aroma primarily develops from precursors through several key pathways: Maillard browning process, lipid degradation, thiamine thermolysis, and cross-talk between Maillard and lipid reaction derivatives [9]. These reactions utilize a diverse pool of hydrophilic precursors, including organic acids, thiamine, oligopeptides, reducing sugars, alongside free amino acids, as well as lipid-soluble ones, such as triglycerides, diglycerides, phospholipids, and free fatty acids. This process generates a broad spectrum of VFCs, including ketones, esters, aldehydes, alcohols, heterocyclic compounds, and hydrocarbons [10]. Among these, aldehydes are particularly important due to their low odor thresholds and characteristic fatty, green, and grassy notes [9]. Fatty acids serve as the central precursors, which are transformed into characteristic meat aroma compounds via enzymatic oxidation, lipid peroxidation, and thermal degradation [11]. Differences in intramuscular fat (IMF) content underlie the characteristic flavor variations observed among pigs [12]. Lipid oxidation is a primary driver of characteristic flavor in beef, where triglycerides and phospholipids rich in unsaturated fatty acids serve as the major aroma precursors [13]. Similarly, specific lipid molecules, particularly certain triglycerides with distinct fatty acid compositions, have been identified as critical contributors to roasted pork odor [14]. Meishan pigs are characterized by a high IMF content, a trait that is strongly associated with flavor potential in pork [15]. However, it remains unclear which specific lipid molecules are primarily responsible for this flavor enhancement and how they are metabolically regulated.

Oxidation of lipids represents the primary pathway for generating VFCs in meat products, encompassing both non‑enzymatic thermal oxidation and enzymatic pathways. Thermal oxidation proceeds through defined mechanistic pathways such as peroxidation and cleavage, whereby saturated and unsaturated fatty acids are transformed into distinct VFCs encompassing ketones, aldehydes, and furans [16, 17]. Among the enzymes involved, lipoxygenases (LOXs) are pivotal, as they mediate the oxidative breakdown of polyunsaturated fatty acids to generate hydroperoxide flavor precursors, which subsequently give rise to key aroma compounds such as alcohols and aldehydes [18, 19]. Within the LOX family, arachidonate 5‑lipoxygenase (*ALOX5*) has been classically defined by its role in inflammation, where it converts arachidonic acid into leukotrienes [20]. Recently, attention has shifted to its ability to oxidize a broader range of fatty acid substrates, including linoleic acid and α‑linolenic acid, leading to aldehyde production [21, 22]. Despite this expanded functional profile, it remains entirely unclear whether *ALOX5* plays any role in pork flavor development, or whether its expression varies among pig breeds in a manner that could explain differences in flavor profiles.

This investigation aimed to systematically compare the volatile flavor fingerprints of five distinct pig breeds and to decipher the molecular basis underlying the characteristic flavor of Meishan pork. First, VFC profiles among Meishan, crossbred and commercial pigs were compared via electronic nose, HS-SPME-GC-MS and relative odor activity value calculation to pinpoint the key flavor compounds. Integrated metabolomics, lipidomics, and weighted correlation network analysis (WGCNA) were then applied to pinpoint associated precursors, with in vitro heating validating their conversion. We additionally performed transcriptomics and WGCNA to uncover regulatory candidates governing precursor accumulation and their subsequent conversion to VFCs. Candidate regulators were then functionally validated by tissue and cellular assays. This multi‑omics and experimental framework provides a mechanistic link between flavor compounds and precursors and a candidate regulatory gene, offering new perspectives on the genetic basis of pork flavor and molecular selection target.

## Materials and Methods

### Animal ethics approval

All animal experimental protocols received ethical validation from the Animal Ethics Committee of Nanjing Agricultural University (approval number: PZ2023052). All sampling and animal handling procedures were carried out in full compliance with the Guidelines for the Ethical Treatment of Experimental Animals (No. 398, 2006) promulgated by the Ministry of Science and Technology of the People’s Republic of China.

### Experimental design and sample collection

In total of 29 pigs from five breeds were included in this study: Meishan (MS, *n* = 5), Yorkshire × Meishan (MY, *n* = 6), Pietrain × Meishan (MP, *n* = 6), Landrace (LR, *n* = 6), and Duroc × Landrace × Yorkshire (DLY, *n* = 6). These five groups were designed to capture a genetic continuum: one purebred Chinese indigenous breed (MS), one purebred commercial breed (LR), two F1 crosses (MY and MP) serving as intermediates between MS and Western breeds, and a commercial three-way cross (DLY). The MS pigs, as a protected indigenous breed, are maintained as a relatively closed breeding population, which is expected to result in relatively high genetic homogeneity. Within each group, animals were selected from separate litters whenever possible to minimize litter‑specific effects, ensuring that familial relatedness did not systematically bias the breed comparisons. All pigs were castrated males with similar ages of approximately 190 d. The animals were raised at the Huaian Institute Experimental Farm of Nanjing Agricultural University in Huaian, Jiangsu Province. All pigs were housed under uniform dietary and environmental conditions, with unrestricted access to drinking water delivered through low-pressure bowl drinkers. A DC variable-frequency floor heating and air conditioning system (Guangdong Alex Eco-conditioning Engineering Co., Ltd., Guangdong, China) was deployed to maintain the barn temperature at 23.55 ± 2.14 °C and relative humidity at 69.51% ± 2.03%. A uniform basal diet was formulated for all animals in compliance with the Nutrient Requirements of Swine (GB/T 39235–2020) [23]. The detailed ingredients and nutrient composition of the diet are summarized in Table S1.

Once the feeding trial reached its endpoint, pigs were humanely slaughtered on the farm by qualified personnel under veterinary supervision and in accordance with animal welfare standards. Prior to slaughter, the pigs were held in a lairage overnight with a 16-h fasting period, during which they had free access to water. Following standard industrial operating procedures and animal welfare protocols, the pigs were anesthetized and then exsanguinated. Within 30 min post-slaughter, *longissimus thoracis* muscle samples were excised from the 6^th^ to 7^th^ rib interval along the dorsal midline. All visible subcutaneous fat and connective tissue were trimmed off immediately. Each specimen was split into two subsamples: one portion was maintained at 4 °C for subsequent meat quality analysis, and the other was rapidly flash-frozen via liquid nitrogen immersion and then stored at −80 °C for prolonged preservation.

### Evaluation of meat quality

Meat characteristic indices were analyzed using *longissimus thoracis* muscle samples collected from the 6^th^–7^th^ rib region. Muscle pH was determined at 45 min post-mortem using a portable auto-temperature-compensating pH meter (Seven2Go™, Mettler Toledo, Greifensee, Switzerland), which was pre-standardized using pH 4.00 and 7.00 standard buffers, as described previously [24]. The final pH value per individual specimen was derived from the mean value across three replicates.

Shear force was assayed with a C-LM3B digital muscle tenderness analyzer (Tenovo International Co., Ltd., Beijing, China). Prior to testing, muscle specimens were incubated within a constant-temperature water tank until the internal core temperature reached 70 °C, held under this thermal condition for 10 min, and then cooled to ambient temperature, as described previously [25]. Each sample was cut parallel to the muscle fiber orientation into 5 cm × 1 cm × 1 cm strips for measurement. For each meat sample, three replicate strips were prepared, and each strip was subjected to three shear tests. The mean value of all replicates served as the final shear force value of the sample.

Crude fat content in muscle tissue was quantified via Soxhlet extraction following the standard method of the National Standards of the People's Republic of China (GB/T 6433–2025) [26].

### Electronic nose analysis

Volatile flavor compound (VFC) profiles were detected using the PEN3 electronic olfactory detection platform (Airsense Analytics GmbH, Schwerin, Germany) fitted with a set of 10 metal oxide semiconductor-based sensing probes; detailed specifications for each sensor are summarized in Table S2. Briefly, approximately 1.0 g of *longissimus thoracis* muscle specimens was transferred into a 10-mL sealed headspace vial. Following 30 min of equilibration at 95 °C, dynamic headspace sampling was conducted. Headspace VFCs were introduced into the sensing chamber with a steady volumetric flow of 400 mL/min over a 150 s detection window, with signal data recorded at a sampling frequency of 1 Hz. Sensor response values within the stable plateau phase (121–125 s) were extracted for subsequent data analysis. After each individual measurement, the system was flushed with purified air for 300 s to restore sensor signals to their baseline level. Each biological sample was assayed in three technical replicates.

### Analysis of VFCs

Frozen muscle specimens preserved at −80 °C were retrieved and pulverized into a fine homogeneous powder, followed by thorough vortex mixing. For each sample and fatty acid standard, an accurately weighed 0.2 g aliquot was transferred to a 20-mL headspace sampling flask. Subsequently, 0.2 g of sodium chloride (Sinopharm Chemical Reagent Co., Ltd., 10019318, Shanghai, China) powder and 20 µL of 2-methyl-3-heptanone (Macklin, M863278-1ml, Shanghai, China) working solution (as internal standard) were loaded into the flask.

VFCs were extracted via automated headspace solid-phase microextraction (HS-SPME). Before each extraction run, the 50/30 µm DVB/CAR/PDMS SPME fiber (57328-U; Supelco, Bellefonte, PA, USA) was pre-conditioned in the GC inlet at 250 °C for 7 min. Each vial was incubated at 95 °C for 5 min to reach equilibration, after which the SPME fiber was inserted into the vial headspace for 30 min to adsorb volatile analytes prior to gas chromatography-mass spectrometry (GC-MS) analysis.

After extraction, the retained compounds were thermally desorbed in the Agilent 8890 GC inlet (Agilent Technologies, Santa Clara, CA, USA) at 250 °C for 5 min in splitless mode. Chromatographic separation was performed on an Agilent 8890 GC system coupled to a 7000E mass spectrometer (Agilent Technologies) using a DB‑5MS capillary column (30 m × 0.25 mm i.d., 0.25 µm film thickness; Agilent J&W, Santa Clara, CA, USA) with 5% phenyl‑polymethylsiloxane as the stationary phase. High‑purity helium was used as the carrier gas at a constant flow rate of 1.2 mL/min, and the inlet temperature was maintained at 250 °C. The oven temperature program was as follows: 40 °C held for 3.5 min, ramped to 100 °C at 10 °C/min, then to 180 °C at 7 °C/min, and finally to 280 °C at 25 °C/min, held for 5 min. The mass spectrometer was operated in electron impact (EI) mode at 70 eV, with the quadrupole, ion source, and transfer line temperatures set at 150, 230, and 280 °C, respectively.

For compound identification, full‑scan acquisition (*m/z* 50–550) was initially performed on representative samples to obtain complete mass spectra. Compounds were identified using an in‑house database built from the NIST library (National Institute of Standards and Technology, Gaithersburg, MD, USA), literature data, and authentic standards where available, with retention index (RI) confirmation using n‑alkane standards (C7–C40) run under identical conditions. A match factor threshold of > 800 and an RI tolerance window of ±20 were applied. Compounds were assigned confidence levels according to Metabolomics Standards Initiative (MSI) criteria: Level 1 for standard‑confirmed compounds and Level 2 for putatively annotated compounds based on MS and RI matching.

For quantitative profiling, based on the compounds identified from full‑scan data, characteristic ions (one quantitative ion and two to three qualitative ions per compound) were selected, and a selected ion monitoring (SIM) method was established. All samples were then analyzed in SIM mode for accurate quantification. Quantification was performed semi‑quantitatively using the internal standard method with 2‑methyl‑3‑heptanone as the internal standard, and results are reported as relative abundances based on peak area ratios to the internal standard.

### Determination of flavor attributes and odor activity values

All identified VFCs were annotated with sensory descriptors by querying authoritative databases (FlavorDB2: https://cosylab.iiitd.edu.in/flavordb2/search; FOODB: https://foodb.ca; The Good Scents Company Information System: https://www.thegoodscentscompany.com; FoodFlavorLab: http://foodflavorlab.cn/#/v2/home; Flavor and Extract Manufacturers Association of the United States: https://www.femaflavor.org). The relative odor activity value (rOAV_*i*_) was determined using the formula: rOAV_*i*_ = C_*i*_/OT_*i*_, where C_*i*_ represents the relative concentration of the compound and OT_*i*_ denotes its odor threshold in water [24].

For flavor profile analysis, all annotated VFCs were classified into five flavor categories primarily based on the FEMA (Flavor and Extract Manufacturers Association) database: (1) Off‑flavor (fishy, pungent, sour); (2) Meaty, savory, sulfurous, almond; (3) Almond, roasted bread; (4) Floral, cheese, fat; and (5) Fruity. Compounds with ambiguous or unassigned flavor descriptors were excluded from this classification. For each sample, the relative content of all compounds within each flavor category was summed, and the proportion of each flavor category was calculated as the percentage of the total relative content of all flavor‑active compounds in that sample. This approach follows the flavor classification strategy previously employed in meat flavor analysis [27].

### Analysis of flavor precursor compounds

Metabolomic analysis: Frozen muscle samples were finely ground under liquid nitrogen. Each 20 mg subsample was extracted using 400 µL pre-chilled methanol–aqueous mixture (7:3, v/v; methanol: Sinopharm Chemical Reagent Co., Ltd., 10014118, Shanghai, China) spiked with internal reference compounds. After vortexing, ice incubation and centrifugation (13,800 × *g*, 10 min, 4 °C), 200 µL of the supernatant was collected for LC-MS analysis. Chromatographic separation was performed on a Waters ACQUITY Premier HSS T3 column (Waters Corporation, Milford, MA, USA) at 40 °C, with 0.1% formic acid in water (A) and acetonitrile (B) as mobile phases at 0.4 mL/min. The gradient was set as: 5%–20% B (2 min), 20%–60% B (3 min), 60%–99% B (1 min), hold 1.5 min, then re-equilibrated to 5% B. Samples were analyzed in both ESI positive and negative modes (4 µL injection) on a TripleTOF system (SCIEX, Framingham, MA, USA) in IDA mode. Key source parameters: GS1/GS2 50 psi, CUR 25 psi, TEM 550 °C; ISVF ± 5,000 V, DP ± 60 V for both modes. Full scans (*m/z* 50–1,000 Da) and product ion scans were acquired. Raw data were computationally processed using the XCMS program to perform peak matching and calibration, and metabolites were annotated through matching against in‑house (MedDB3), public (HMDB, KEGG, MoNA, MassBank), and AI‑assisted (Insilico 1.2, a predicted spectral library built using a non‑public in‑house database as the training set) databases. Annotations were classified into confidence levels 1a–4: 1a (high‑confidence match based on standard, MS1, RT, and MS2), 1b (moderate‑confidence match based on standard, MS1, RT, and MS2), 2 (high‑confidence match based on MS1, RT, and MS2), 3 (moderate‑confidence match based on MS1, RT, and MS2), and 4 (match based on MS1 and RT only). Only levels 1a–3 were retained for differential analysis. When conflicts occurred, priority was given to in‑house database matches, followed by public databases and the AI‑assisted database, with the highest scoring annotation selected within the same database. According to MSI criteria, levels 1a and 1b correspond to MSI Level 1, level 2 to MSI Level 2–3 (higher score), and level 3 to MSI Level 2–3 (lower score).

Profiling of fatty acid constituents: Total lipids were isolated from muscle tissue via an adjusted Folch lipid extraction protocol, as described previously [24]. A 2-g sample of *longissimus thoracis* was weighed and transferred into a 15-mL tube, spiked with 200 µL heptadecanoic acid (C17:0, 10 mg/mL in methanol; Sigma‑Aldrich, H3500, St. Louis, MO, USA) serving as the internal reference compound, then homogenized with 2 mL chloroform/methanol (2:1, v/v; chloroform: Sinopharm Chemical Reagent Co., Ltd., 10006862, Shanghai, China; methanol: Sinopharm Chemical Reagent Co., Ltd., 10014118, Shanghai, China). After 1 mL of 8.3 mol/L hydrochloric acid (Sinopharm Chemical Reagent Co., Ltd., 10011062, Shanghai, China) was supplemented, the blended suspension was hydrolyzed at 80 °C for 2 h. After the system was cooled to room temperature, 1 mL 2% (w/v) sodium hydroxide (Sinopharm Chemical Reagent Co., Ltd., 100197630, Shanghai, China) was supplemented for saponification at 65 °C for 30 min, followed by methylation with 2 mL 14%–15% (w/v) boron trifluoride-methanol solution (Meryer, M33824-500ML, Shanghai, China) over a 30-min incubation at 65 °C. After the system was cooled to room temperature, 2 mL saturated sodium chloride solution and 2 mL n-hexane (Sinopharm Chemical Reagent Co., Ltd., 80068618, Shanghai, China) were blended. After 2 min vortexing and 5 min centrifugation at 1,000 × *g*, hexane supernatant enriched with fatty acid methyl esters (FAMEs) was harvested, passed through a 0.22-μm PTFE filter membrane (Millex-GP, Millipore, SLGP033RB, Burlington, MA, USA), and then measured with an Agilent 6890 N GC coupled with 5977 MS (Agilent Technologies). Separation was carried out utilizing a CP-Sil 88 capillary column (100 m × 0.25 mm × 0.25 µm; Agilent J&W) with helium carrier gas at 1.0 mL/min, split ratio 10:1, injection volume 1 µL. The column temperature gradient schedule was: 100 °C, 5 min, ramp to 240 °C at 4 °C/min, 15 min. FAMEs were annotated and quantified via the internal standard method, using a validated 37-blend FAME reference mixture (Supelco 18919‑1AMP, Sigma‑Aldrich).

### Transcriptomic analysis

Transcriptome sequencing of *longissimus thoracis* samples was carried out by LC-Bio Technology Co., Ltd. (Hangzhou, China). Total RNA was extracted from tissue samples via TRIzol lysis reagent (Vazyme Biotech Co., Ltd., R401-01, Nanjing, China), and RNA quality was evaluated across three metrics: concentration (Nanodrop ND-1000; Thermo Fisher Scientific, Waltham, MA, USA), integrity (Agilent 2100 Bioanalyzer, RIN > 7.0), and total yield (> 1 μg). Sequencing libraries were constructed from qualified RNA via a standard workflow including oligo(dT) mRNA enrichment, fragmentation, strand-specific cDNA synthesis, adapter ligation, and PCR amplification. The constructed sequencing libraries featuring a mean fragment length of 300 ± 50 bp were profiled via an Illumina NovaSeq 6000 sequencing system (Illumina, Inc., San Diego, CA, USA) using the 150 bp dual-end read (PE150) mode.

Clean sequence data were aligned against the reference genome, and transcript abundance levels were calculated via the transcripts-per-million (TPM) normalization metric. Differential gene expression analysis was subsequently conducted on raw read count data using the DESeq2 package (v1.38.3; Bioconductor, https://bioconductor.org), with significance cutoffs defined as |log_2_(fold change)| > 1 and adjusted *P*-value (*P*_adj_) < 0.05.

### Weighted correlation network analysis

To systematically identify molecular features associated with the formation of key flavor compounds, WGCNA was carried out utilizing both metabolomic/lipidomic and transcriptomic data, following an established integrative omics framework [28]. All computational workflows were run within R (v4.2.2) via the WGCNA package (v1.71), with the relative abundances of the key flavor compounds defined as the phenotypic traits for correlation.

To select the optimal soft‑thresholding power, candidate powers from 1 to 20 were evaluated using the pickSoftThreshold function in the WGCNA package. The power was chosen at the elbow point where the scale‑free topology fit index (*R*^2^) began to plateau, with an *R*^2^ threshold of > 0.8. Based on this criterion, a power of 16 was selected for the metabolomic/lipidomic dataset and 13 for the transcriptomic dataset.

For the identification of potential flavor precursors, the combined quantitative dataset of all metabolites and fatty acids from the 29 *longissimus thoracis* samples served as the input matrix. A soft-thresholding power of 16 was applied to construct a signed network. Modules of highly correlated features were detected using dynamic tree cutting, with a minimum module size of 10 and a merge cut height of 0.25. Metabolites within significantly associated modules were considered candidate hub precursors if they met the dual criteria of strong intramodular connectivity (module membership |MM| > 0.7, *P* < 0.05) and a high correlation with the flavor trait (gene significance |GS| > 0.4, *P* < 0.05).

For the identification of key regulatory genes, the gene expression matrix from transcriptomic sequencing of the same 29 samples was used as input. A separate signed co-expression network was constructed with a soft-thresholding power of 13. Gene modules were identified with a minimum size of 20 and merged at a height cut of 0.15. Genes within key modules were prioritized as candidate regulators based on their high module membership and strong correlation with the flavor traits.

### Cell culture and treatment

The C2C12 mouse myoblast cell line was propagated in Dulbecco’s modified Eagle’s medium (DMEM; Wisent Inc., 319-005-CL, Nanjing, China) fortified with 10% fetal bovine serum (Gibco, Thermo Fisher Scientific, 10099141, Australia) and 100 IU/mL penicillin–streptomycin (Wisent Inc., 450-201). All cultures were kept at 37 °C in a humidified incubator under 5% CO₂. Cells at the exponential growth stage were seeded into 6-well culture plates. Once cells reached approximately 80%–90% confluence, they were exposed to oleic acid (OA; Sigma-Aldrich, O1008-1G) or α-linolenic acid (ALA; Sigma-Aldrich, L2376-500MG) for a 24-h treatment period, after which samples were collected for downstream analysis.

Fatty acid–BSA conjugates were prepared via a saponification-complexation method. Briefly, OA and ALA were saponified in 0.1 mol/L NaOH at 75 °C to yield 20 mmol/L sodium salt solutions. The clarified solutions were immediately mixed 1:1 (v/v) with 20% (w/v) fatty acid-free BSA (Beyotime Biotechnology, ST023, Shanghai, China) prepared in PBS. After cooling to room temperature, the mixture was sterile-filtered through a 0.22-µm membrane filter (Millex-GP, Millipore, SLGP033RB, Burlington, MA, USA) and stored at 4 °C. The final concentration of each fatty acid in the complexes was 10 mmol/L.

The overexpression plasmid for *ALOX5* and the corresponding empty vector control were designed and synthesized by Wuhan Miaoling Biotechnology Co., Ltd. (Wuhan, China). A specific small interfering RNA (siRNA) targeting *ALOX5* and a scrambled siRNA control were synthesized by Tsingke Biotechnology Co., Ltd. (Nanjing, China). The siRNA sequences were as follows: sense strand, 5'-CGAGCGCAAUAAGAACAAA(dT)(dT)-3'; antisense strand, 5'-UUUGUUCUUAUUGCGCUCG(dT)(dT)-3'. C2C12 cells were transfected with the *ALOX5* overexpression plasmid or the *ALOX5*-specific siRNA using JetPRIME^®^ transfection reagent (Polyplus Transfection, Illkirch, France). The empty vector and scrambled siRNA were used as negative controls for the overexpression and knockdown experiments, respectively.

### Aldehyde compound content assay

Fluorimetric determination of total aldehyde content was carried out using the Amplite® Aldehyde Quantitation Kit (Cat. No. 10052, AAT Bioquest, Sunnyvale, CA, USA) in accordance with the manufacturer’s protocol. Samples (0.1 g of *longissimus thoracis* tissue or C2C12 cell pellets) were first homogenized in ice-cold PBS, then centrifuged at 10,000 × *g* for 10 min at 4 °C. Aldehyde standard solution (0–1,000 μmol/L) or 50 μL aliquot of the supernatant was combined with 50 μL of AldeLight™ Blue working solution. The mixture was transferred to an opaque black 96-well plate, incubated for 15 min at room temperature in the dark, and then mixed with 25 μL of reaction buffer. Fluorescence intensity was detected on a Synergy H1 microplate reader (BioTek, Winooski, VT, USA) with excitation and emission wavelengths set to 365 nm and 435 nm, respectively.

### Total RNA extraction and quantitative real-time PCR

Total RNA was extracted using TRIzol reagent. The concentration and purity of the RNA were determined using a Nanodrop 1000 spectrophotometer. Subsequently, 1 µg of total RNA was reverse transcribed into complementary DNA (cDNA) using a Vazyme reverse transcription pre-mix kit (R323-01; Vazyme Biotech Co., Ltd., Nanjing, China) according to the manufacturer's instructions. Quantitative PCR (qPCR) amplification was performed on a Stratagene Mx3000P qPCR system (Agilent Technologies) using 2 µL of 1:20 diluted cDNA in a total reaction volume of 10 µL, with specific primers for each target gene (sequences listed in Table S3). The qPCR cycling parameters were set as follows: initial denaturation at 95 °C for 30 s, followed by 40 cycles of 95 °C for 5 s and 60 °C for 30 s. The housekeeping gene PPIA served as the internal reference for normalization, as it exhibited stable expression across all experimental groups. All gene-specific primers were commercially synthesized by Tsingke Biotechnology Co., Ltd. (Beijing, China). Gene expression levels were computed via the 2^−^^ΔΔCt^ method.

### Total protein extraction and Western blot analysis

Tissue samples and cell samples were homogenized in RIPA lysis buffer (Beyotime Biotechnology, P0013B, Shanghai, China) containing protease inhibitors. The protein concentration of the lysates was determined using a BCA protein assay kit (TransGen Biotech Co., Ltd., DQ111-01, Beijing, China). 20–40 µg of total protein per sample was loaded and separated on a 10% polyacrylamide gel. The separated proteins were then transferred onto a nitrocellulose membrane (Cat. No. 1620112; Bio-Rad Laboratories, Hercules, CA, USA) using a wet transfer system. The membrane was blocked and subsequently incubated at 4 °C with the primary antibodies listed in Table S4. After completion of the washing procedure, the membrane was probed with a host-appropriate secondary antibody conjugated to horseradish peroxidase (HRP). Protein bands were visualized using an enhanced chemiluminescence (ECL) substrate solution (Biosharp, BL520A, Guangzhou, China) and imaged on a Tannon-5500 gel documentation system (Tanon Science & Technology Co., Ltd., Shanghai, China). Band density was quantified with ImageJ software (v1.53; National Institutes of Health, Bethesda, MD, USA), with α-tubulin used as the internal loading control.

### Statistical analysis

Quantitative data are presented as mean ± standard error of the mean (SEM). Statistical analyses were performed using SPSS 22.0 (IBM Corp., Armonk, NY, USA) and GraphPad Prism v8.0 (GraphPad Software, San Diego, CA, USA). Differences between two groups were assessed using Student's *t*-test, while comparisons among more than two groups were conducted by one-way analysis of variance (ANOVA). To ensure statistical robustness, Levene's test was conducted, confirming that the assumption of homogeneity of variances was satisfied across all groups. Pearson correlation analysis was performed using SPSS software (version 22.0). A *P*-value of less than 0.05 was considered statistically significant for all analyses.

For transcriptomic data, differential expression was analyzed using DESeq2 with an adjusted *P*-value (*P*_adj_) < 0.05 (Benjamini–Hochberg FDR). For metabolomic and lipidomic data, differential features were identified using ANOVA or *t*-test, followed by Benjamini–Hochberg FDR correction (*q* < 0.05).

## Results

### Volatile flavor profiles across five pig breeds

**Fig. 1:**
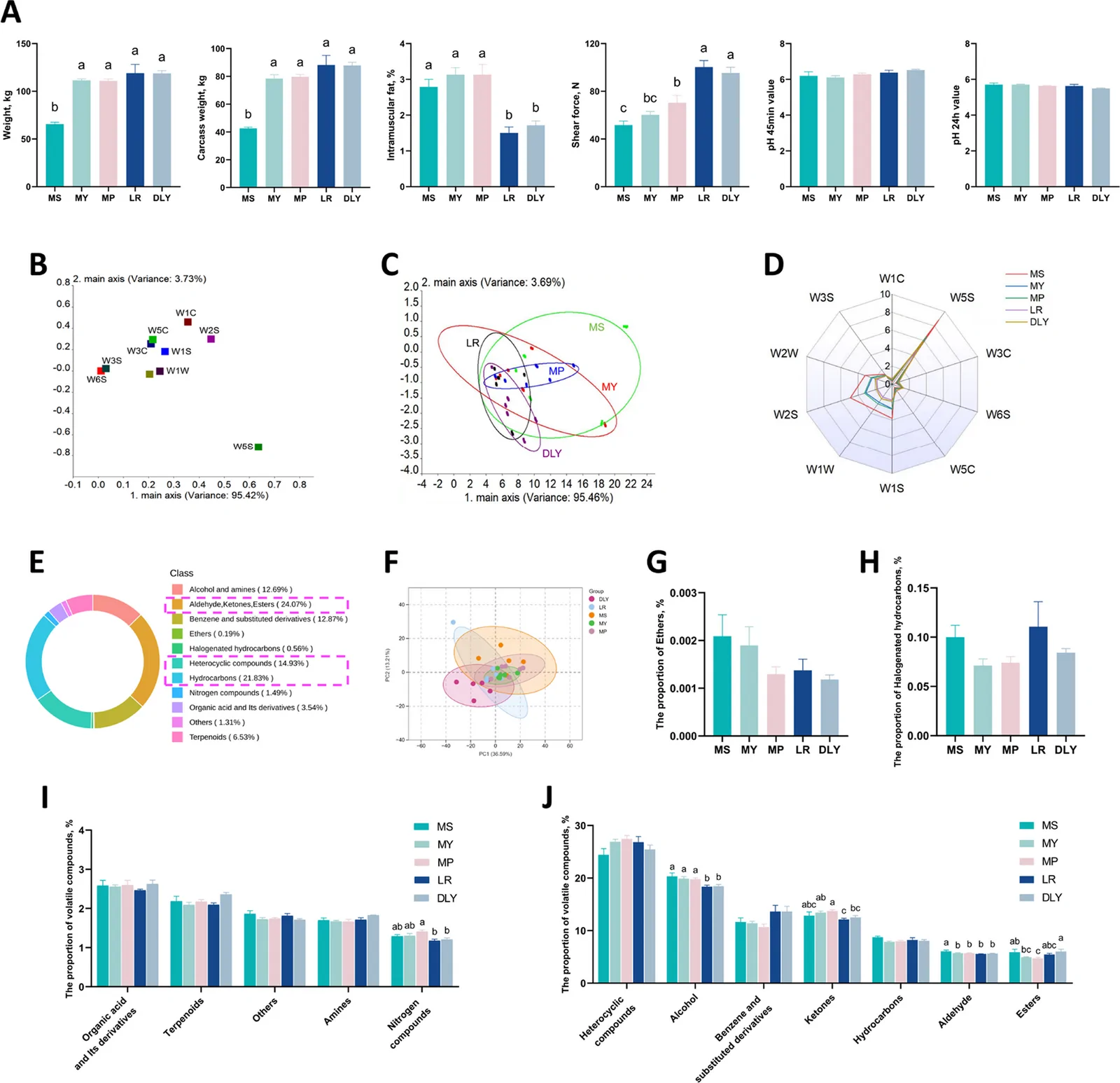
Volatile flavor profiles across five pig breeds. **A** Evaluations of live weight, carcass mass and meat quality characteristics in five pig varieties. **B** Loading graph for the electronic nose MOS sensor array. Every plotted point stands for an independent metal oxide semiconductor sensor. The distance between each sensor coordinate and the origin signifies its weight in principal component analysis: sensors with larger offsets from the origin deliver greater discriminatory power across samples. **C** PCA (principal component analysis) for E-nose analysis of five groups. **D** Radar chart of E-nose sensor response patterns. **E** Composition of VFCs classes. **F** Principal component analysis (PCA) of volatile profiles. **G–J** Comparison of relative VFCs class abundances. Data are mean ± SEM (*n* = 5 or 6). Different letters denote significant differences (*P* < 0.05) among groups

As a distinctive Chinese indigenous breed, MS pigs exhibited significantly lower body weight (*P* < 0.01) and carcass weight (*P* < 0.01) relative to the remaining four swine varieties. The IMF content was higher in MS pigs than in the introduced commercial breeds. Notably, the crossbred pigs (MY and MP) also possessed higher IMF content than the commercial breeds (LR and DLY) (*P* < 0.01). Furthermore, MS pork demonstrated the best tenderness among all groups (*P* < 0.01). However, no statistically significant differences were detected for muscle pH values at 45 min and 24 h post-mortem across the five groups (Fig. 1A).

To detect VFCs, a preliminary analysis was conducted using an electronic nose (E-nose) on heated meat samples from all groups. The overall odor profile was predominantly characterized by nitrogen oxides (W5S), aldehydes/alcohols/ketones (W2S), and aromatic compounds (W1C; Fig. 1B). Although the discriminatory power of the electronic nose was limited, as indicated by the lack of clear separation among the five groups in the principal component analysis (PCA; Fig. 1C), the response values of six specific sensors (W1C, W3C, W5C, W1S, W2S, and W3S) differed significantly (*P* < 0.05; Fig. 1D and Fig. S1A). The LR group produced the maximal signal outputs from sensors W1C, W3C, and W5C, which exhibit strong responsiveness to aromatic compounds, ammonia, and short-chain alkanes, respectively (*P* < 0.05). These responses displayed markedly greater signal intensity than those in the MS and MP groups. In contrast, the MS group exhibited the highest responses for sensors W1S, W2S, and W3S (*P* < 0.05), which are primarily sensitive to methane, aldehydes/alcohols/ketones and long-chain alkanes, respectively, and these signal intensities were markedly higher than those in the LR and DLY groups.

The specific VFCs were detected and quantified via HS-SPME-GC-MS. A total of 542 VFCs were annotated. In terms of the number of compounds detected, the three most abundant categories were aldehydes/ketones/esters, hydrocarbons, and heterocyclic compounds (Fig. 1E), which was generally in line with the E-nose findings. Similarly, PCA of the HS-SPME-GC-MS data revealed no distinct separation among the five breed groups (Fig. 1F). The relative content of each chemical class, expressed as a percentage of the total detected VFCs, was compared. No statistically significant differences were observed among experimental cohorts for ethers, terpenoids, amines, hydrocarbons, heterocyclic compounds, organic acids and their derivatives, and benzene and substituted derivatives (Fig. 1G–J). In contrast, significant inter-breed differences were found for alcohols, ketones, aldehydes, esters, and nitrogen compounds. Specifically, the MP group showed the highest content of ketones and nitrogen compounds, which were markedly elevated compared with those quantified in the LR as well as DLY experimental cohorts (*P* < 0.05; Fig. 1I and J). The DLY group exhibited the highest esters content, significantly surpassing the MP and MY groups (*P* < 0.05). Furthermore, the alcohol content was higher in the MS, MY, and MP groups than in the LR and DLY groups (*P* < 0.01). Most notably, the relative abundance of aldehydes was highest in the MS group and was significantly greater than that in the other four groups (*P* < 0.05; Fig. 1J).

### Flavor profiles and characteristic flavor compounds of Meishan pork

**Fig. 2:**
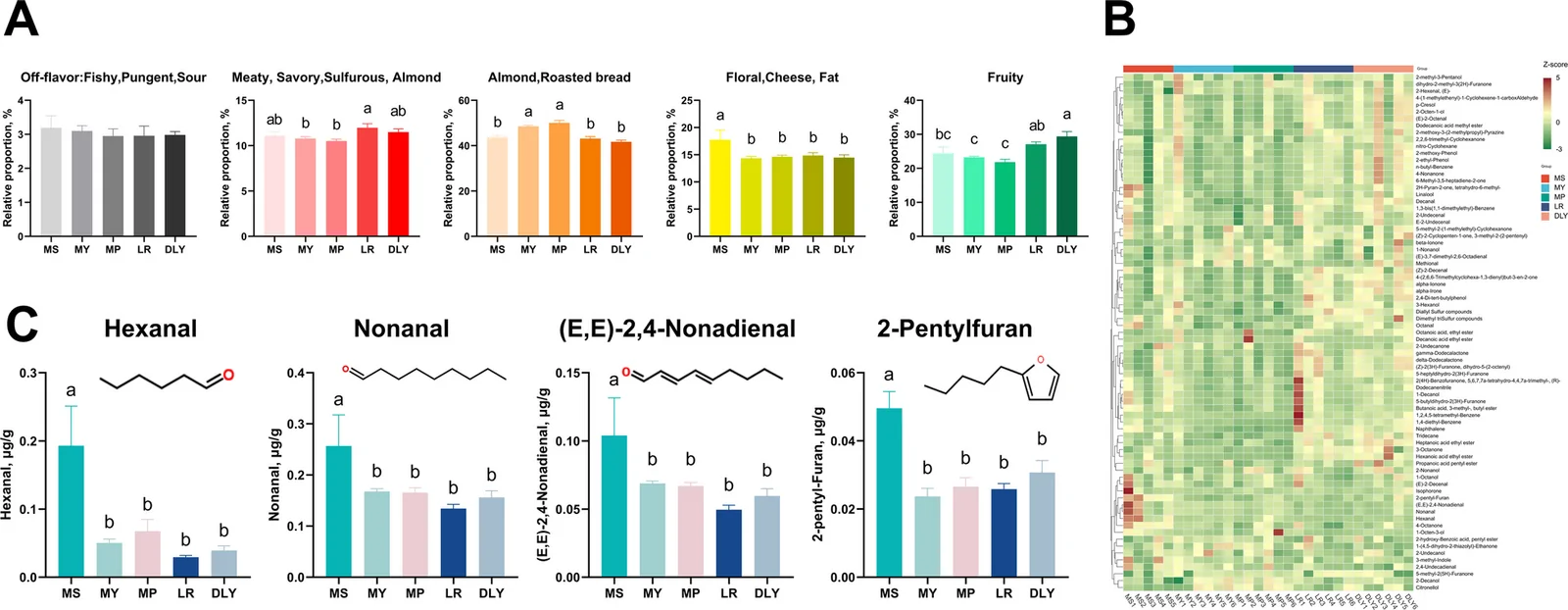
Flavor profiles and characteristic flavor compounds of Meishan pork. **A** Flavor category profiles of five pig breeds. Volatile compounds were classified into five flavor categories based on sensory descriptors, and the relative content of each category is expressed as a percentage of the total flavor‑active compounds. **B** Heatmap of relative odor activity values (rOAV > 1) for 75 key flavor compounds across five pig breeds. **C** Comparison of the relative content of the four characteristic flavor compounds. Data are mean ± SEM (*n* = 5 or 6). Different letters denote significant differences (*P* < 0.05) among groups

To compare the overall flavor profiles across the five breeds, all annotated VFCs were classified into five flavor categories based on their sensory descriptors: (1) Off‑flavor (fishy, pungent, sour); (2) Meaty, savory, sulfurous, almond; (3) Almond, roasted bread; (4) Floral, cheese, fat; and (5) Fruity. The classification of compounds and their relative contents are provided in Additional file 2. For off-flavor compounds, no significant differences were observed among the five breeds. For meaty, savory, sulfurous, and almond-like compounds, LR exhibited the highest relative content, significantly higher than MY and MP (*P* < 0.05), while MS and DLY showed no significant differences from any other groups. For almond and roasted bread-like compounds, MY and MP showed significantly higher levels than MS, LR, and DLY (*P* < 0.05), with no significant difference between MY and MP. For floral, cheese, and fat-like compounds, MS exhibited the highest relative content among all groups (*P* < 0.05), while the other four breeds showed no significant differences among themselves. For fruity compounds, DLY exhibited the highest relative content, significantly higher than MS, MY, and MP (*P* < 0.05); LR showed intermediate levels, with no significant difference from DLY or MS, but significantly higher than MY and MP (*P* < 0.05); MY and MP had the lowest levels, with no significant difference between them (Fig. 2A).

The relative odor activity value (rOAV) was calculated for all detected compounds. A total of 75 compounds exhibited an rOAV greater than 1 (Fig. 2B, Table S5), indicating their significant role in pork flavor formation. Among these, only those also present at significantly higher levels in Meishan pork than in the other four breeds (ANOVA,* P* < 0.05) were selected as characteristic flavor compounds. Four compounds—hexanal, nonanal, (E,E)-2,4-nonadienal, and 2-pentylfuran—were identified as characteristic of Meishan pork. The relative contents of these four key flavor compounds were markedly elevated within MS pork compared with the other four breed groups (*P* < 0.05; Fig. 2C). Hexanal is described as having grass, green, and fat-like odors; nonanal imparts citrus, orange, and peel notes; (E,E)-2,4-nonadienal contributes fatty, waxy, green, and fruity aromas; and 2-pentylfuran presents a complex profile including fishy, roasted, popcorn, beany, fruity, green, metallic, buttery, and caramel-like notes.

### Identification of key precursors for characteristic flavor compounds in Meishan pork via WGCNA

**Fig. 3:**
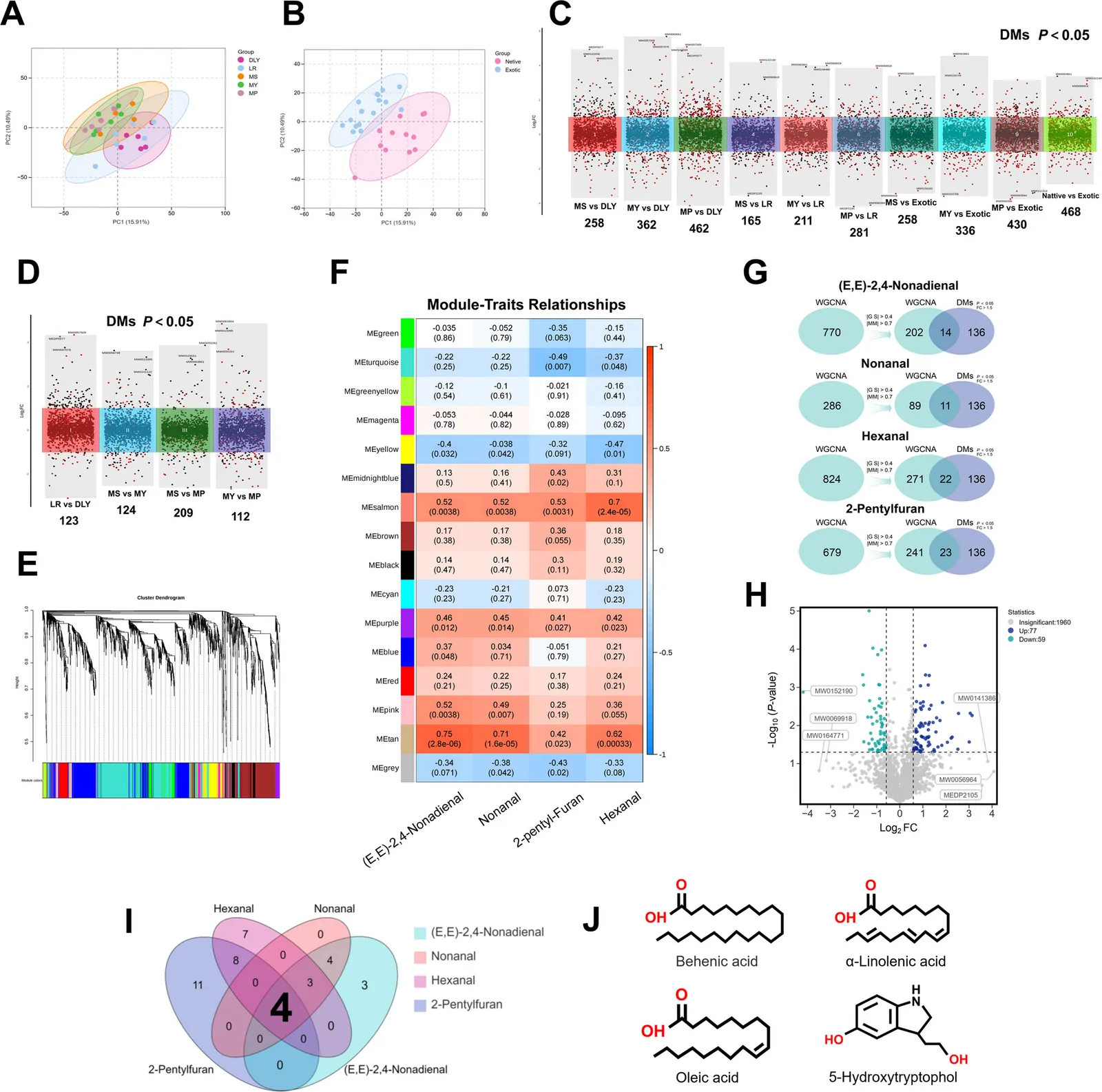
Identification of key precursors for characteristic flavor compounds in Meishan pork via WGCNA. **A** Principal component analysis (PCA) of the intramuscular metabolome across the five groups. **B** PCA of the metabolome grouped by genetic lineage. **C** Volcano plots of differentially abundant metabolites between genetically distant breed pairs. **D** Volcano plots of differentially abundant metabolites between closely related breed pairs. **E** Clustering dendrogram of metabolites from WGCNA. Each branch tip represents a single metabolite, and each color band below the dendrogram denotes a distinct module. **F** Module—trait correlation heatmap. Each cell shows the correlation coefficient and significance. **G** Schematic of the stepwise identification of candidate flavor precursors. **H** Volcano plot of integrated metabolomic and lipidomic profiles between Meishan and Landrace pigs. **I** Identification of shared metabolic precursors. Venn diagram showing the intersection of candidate precursor metabolites for the four characteristic flavor compounds. **J** Chemical structures of the four shared precursor metabolites

To identify the metabolic precursors of the characteristic flavor compounds, we first profiled the intramuscular metabolome of the five pig breeds. PCA of these data revealed no distinct clustering by breed (Fig. 3A). However, when the samples were categorized by genetic lineage—Native (encompassing the indigenous Meishan and its crossbreeds MY and MP) versus Exotic (the introduced LR and DLY breeds)—a distinct separation between these two groups was observed (Fig. 3B). Pairwise comparisons between groups were conducted to identify differential metabolites. The number of differential metabolites between each Native and Exotic breed pair was substantially higher (ranging from 165 to 468; Fig. 3C). In contrast, comparisons within the Native group or within the Exotic group yielded far fewer differential metabolites (ranging from 112 to 209; Fig. 3D). Given that the untargeted metabolomics platform primarily captured hydrophilic metabolites, this analysis was complemented by targeted quantification of fatty acids, which are key precursors of lipid-derived VFCs. Almost all fatty acids measured showed significant variation in their levels among the five groups (*P* < 0.05), with the exception of C18:2n6 and C18:3n6 (Table 1). A consistent trend was observed where MS pigs and their crossbreds (MY and MP) exhibited higher levels of most fatty acids, aligning with their elevated intramuscular fat content. An exception to this trend was C14:0 (myristic acid), with its highest level found in the DLY group. Notably, the N6/N3 polyunsaturated fatty acid ratio was significantly lower in the native lineage pigs (MS, MY, MP) compared with the exotic breeds (LR, DLY) (*P* < 0.01).

**Table 1:** Fatty acids profile of the *longissimus thoracis* muscle in various swine varieties, mg/100 g

Trait	MS	MY	MP	LR	DLY	SEM	*P*-value
Saturated fatty acid							
C14:0	12.59 ± 4.85^c^	13.78 ± 5.48^bc^	19.29 ± 2.91^bc^	19.99 ± 9.20^b^	35.82 ± 2.22^a^	1.84	< 0.0001
C16:0	718.14 ± 144.32^a^	553.27 ± 48.71^c^	660.99 ± 45.93^ab^	566.75 ± 81.49^c^	599.48 ± 41.84^bc^	17.61	0.0099
C17:0	6.11 ± 1.05^a^	5.35 ± 0.70^a^	6.73 ± 0.77^a^	6.37 ± 2.48^a^	3.59 ± 1.00^b^	0.32	0.0051
C18:0	443.61 ± 103.70^a^	337.15 ± 23.81^b^	381.87 ± 20.29^b^	328.9 ± 17.18^b^	344.27 ± 11.32^b^	10.97	0.0022
C20:0	12.66 ± 5.01^a^	9.40 ± 2.14^bc^	12.45 ± 1.02^ab^	11.12 ± 2.31^abc^	8.06 ± 1.14^c^	0.56	0.0256
C22:0	61.16 ± 6.70^a^	46.19 ± 4.91^b^	42.75 ± 3.87^b^	31.56 ± 3.47^c^	21.59 ± 4.70^d^	2.61	< 0.0001
C23:0	1.07 ± 0.37^abc^	1.33 ± 0.20^a^	1.17 ± 0.12^ab^	0.91 ± 0.21^bc^	0.85 ± 0.16^c^	0.05	0.0062
C24:0	13.32 ± 2.84^b^	20.63 ± 1.70^a^	18.39 ± 2.47^a^	8.92 ± 1.17^c^	13.00 ± 1.08^b^	0.87	< 0.0001
SFA	1,268.65 ± 254.33^a^	987.11 ± 76.19^c^	1,143.64 ± 66.88^ab^	974.51 ± 99.17^c^	1,026.66 ± 52.55^bc^	29.57	0.0030
Monounsaturated fatty acid							
C16:1n7	139.99 ± 58.79^a^	92.02 ± 28.00^bc^	119.57 ± 27.00^ab^	55.29 ± 17.64^c^	93.73 ± 22.89^bc^	7.72	0.0031
C17:1n7	3.51 ± 0.80^bc^	4.36 ± 0.84^ab^	5.39 ± 0.88^a^	5.23 ± 0.72^a^	2.69 ± 2.40^c^	0.30	0.0075
C18:1n9	1,322.60 ± 279.53^a^	891.04 ± 165.58^b^	1,238.56 ± 109.66^a^	781.59 ± 117.36^b^	924.55 ± 119.44^b^	48.24	< 0.0001
C24:1n9	6.25 ± 2.15^a^	4.72 ± 0.42^bc^	5.51 ± 0.69^ab^	4.08 ± 0.38^c^	3.59 ± 0.55^c^	0.25	0.0012
MUFA	1,472.34 ± 338.1^a^	992.13 ± 190.17^b^	1,369.04 ± 130.18^a^	846.20 ± 132.10^b^	1,024.56 ± 141.41^b^	55.52	< 0.0001
Polyunsaturated fatty acid							
C18:2n6	240.56 ± 36.52	247.77 ± 21.45	264.11 ± 6.97	236.4 ± 25.74	241.77 ± 17.01	4.36	0.2889
C18:3n6	4.28 ± 1.61	5.31 ± 0.81	4.97 ± 0.88	4.63 ± 0.83	4.45 ± 1.22	0.20	0.5244
C18:3n3	20.00 ± 2.71^a^	15.49 ± 2.39^b^	18.96 ± 2.91^a^	10.91 ± 1.07^c^	11.87 ± 1.36^c^	0.78	< 0.0001
C20:2n6	15.32 ± 5.23^a^	10.30 ± 1.71^bc^	9.76 ± 1.40^bc^	8.18 ± 1.65^c^	12.84 ± 6.62^ab^	0.82	0.0466
C20:3n6	17.99 ± 3.79^a^	15.31 ± 1.20^b^	15.16 ± 1.39^b^	10.36 ± 1.32^c^	10.42 ± 0.82^c^	0.65	< 0.0001
C20:3n3	21.97 ± 8.06^a^	14.77 ± 2.20^bc^	18.04 ± 8.44^ab^	13.67 ± 3.75^bc^	8.41 ± 3.12^c^	1.28	0.0074
C20:4n6	35.86 ± 7.35^a^	28.77 ± 2.34^c^	30.06 ± 2.31^bc^	31.67 ± 2.65^abc^	34.35 ± 2.39^ab^	0.81	0.0225
C20:5n3	6.92 ± 1.93^a^	6.42 ± 0.70^a^	6.44 ± 0.54^a^	5.67 ± 1.21^a^	3.77 ± 0.75^b^	0.28	0.0005
C22:6n3	11.52 ± 5.78^a^	12.87 ± 3.92^a^	12.69 ± 2.97^a^	6.19 ± 2.22^b^	5.83 ± 0.71^b^	0.84	0.0013
PUFA	374.43 ± 26.43^a^	357.00 ± 21.38^ab^	380.20 ± 16.98^a^	327.67 ± 21.78^c^	333.69 ± 13.76^bc^	5.29	0.0003
n3	60.42 ± 12.37^a^	49.54 ± 5.12^b^	56.14 ± 9.80^ab^	36.43 ± 4.46^c^	29.87 ± 3.99^c^	2.54	< 0.0001
n6	298.69 ± 33.59	297.16 ± 22.08	314.30 ± 6.65	283.06 ± 25.06	290.98 ± 17.16	4.29	0.2004
n6/n3	5.25 ± 1.97^c^	6.06 ± 0.88^c^	5.71 ± 0.77^c^	7.90 ± 1.42^b^	9.89 ± 1.51^a^	0.40	< 0.0001
UFA	1,846.76 ± 321.19^a^	1,349.14 ± 196.06^b^	1,749.23 ± 126.49a	1,173.86 ± 132.93^b^	1,358.25 ± 142.27^b^	58.04	< 0.0001
TFA	3,115.42 ± 559.91^a^	2,336.25 ± 265.95^b^	2,892.87 ± 132.27^a^	2,148.38 ± 187.91^b^	2,384.91 ± 188.68^b^	84.21	< 0.0001

The values are presented as means ± standard deviations (*n* = 5/6). ^a^^–^^c^Mean values with different superscript letters differ significantly (*P* < 0.05)

*SFA* Saturated fatty acids = C14:0 + C16:0 + C17:0 + C18:0 + C20:0 + C22:0 + C23:0 + C24:0

*MUFA* Monounsaturated fatty acids = C24:1n9 + C18:1n9 + C17:1n7 + C16:1n7

*PUFA* Polyunsaturated fatty acids = C22:6n3 + C20:5n3 + C20:4n6 + C20:3n3 + C20:3n6 + C20:2n6 + C18:3n3 + C18:3n6 + C18:2n6

n-3 PUFA = C22:6n3 + C20:5n3 + C20:3n3 + C18:3n3

n-6 PUFA = C20:4n6 + C20:3n6 + C20:2n6 + C18:3n6 + C18:2n6

*TFA* Total fatty acids = PUFA + MUFA + SFA

Using the integrated metabolomic and fatty acid datasets as the input matrix, we performed WGCNA, with the concentrations of the four characteristic flavor compounds of MS pork set as phenotypic traits. This analysis identified 15 co-abundance modules (Fig. 3E), with sizes of 590, 484, 353, 158, 104, 90, 70, 52, 46, 39, 31, 20, 17, 17, and 13 metabolites, respectively (Fig. S2A). Module–trait correlation analysis revealed distinct association patterns: (E,E)-2,4‑nonadienal correlated significantly with the yellow, salmon, purple, blue, pink, and tan modules; nonanal with the yellow, salmon, purple, pink, and tan modules; hexanal with the turquoise, yellow, salmon, purple, and tan modules; and 2‑pentylfuran with the turquoise, midnightblue, salmon, purple, and tan modules (Fig. 3F). The relevant modules initially contained 770, 286, 824, and 679 metabolites, respectively. Applying stricter thresholds (|GS|> 0.4 and |MM|> 0.7) refined these to 202, 89, 271, and 241 candidate precursor metabolites (Fig. 3G). Differential metabolite analysis between MS and LR pigs, the breeds with the most divergent levels of the characteristic flavor compounds, identified 136 differential metabolites (*P* < 0.05; Fig. 3H). Intersecting the WGCNA-derived candidate lists with these differential metabolites further narrowed the potential precursors to 14, 11, 22, and 23 metabolites for each flavor compound, respectively (Fig. 3G). Behenic acid (C22:0), α‑linolenic acid (C18:3n3), oleic acid (C18:1n9), and 5‑hydroxytryptophol were the four metabolites common to all refined precursor sets (Fig. 3I and J). Given their prominence within the shared precursor pool and the established pathway of lipid oxidation-derived aldehyde formation, oleic acid and α‑linolenic acid emerged as the key candidates for exploring the metabolic underpinnings of the distinctive MS pork flavor.

### Thermal-oxidative generation of characteristic aldehydes from unsaturated fatty acids

**Fig. 4:**
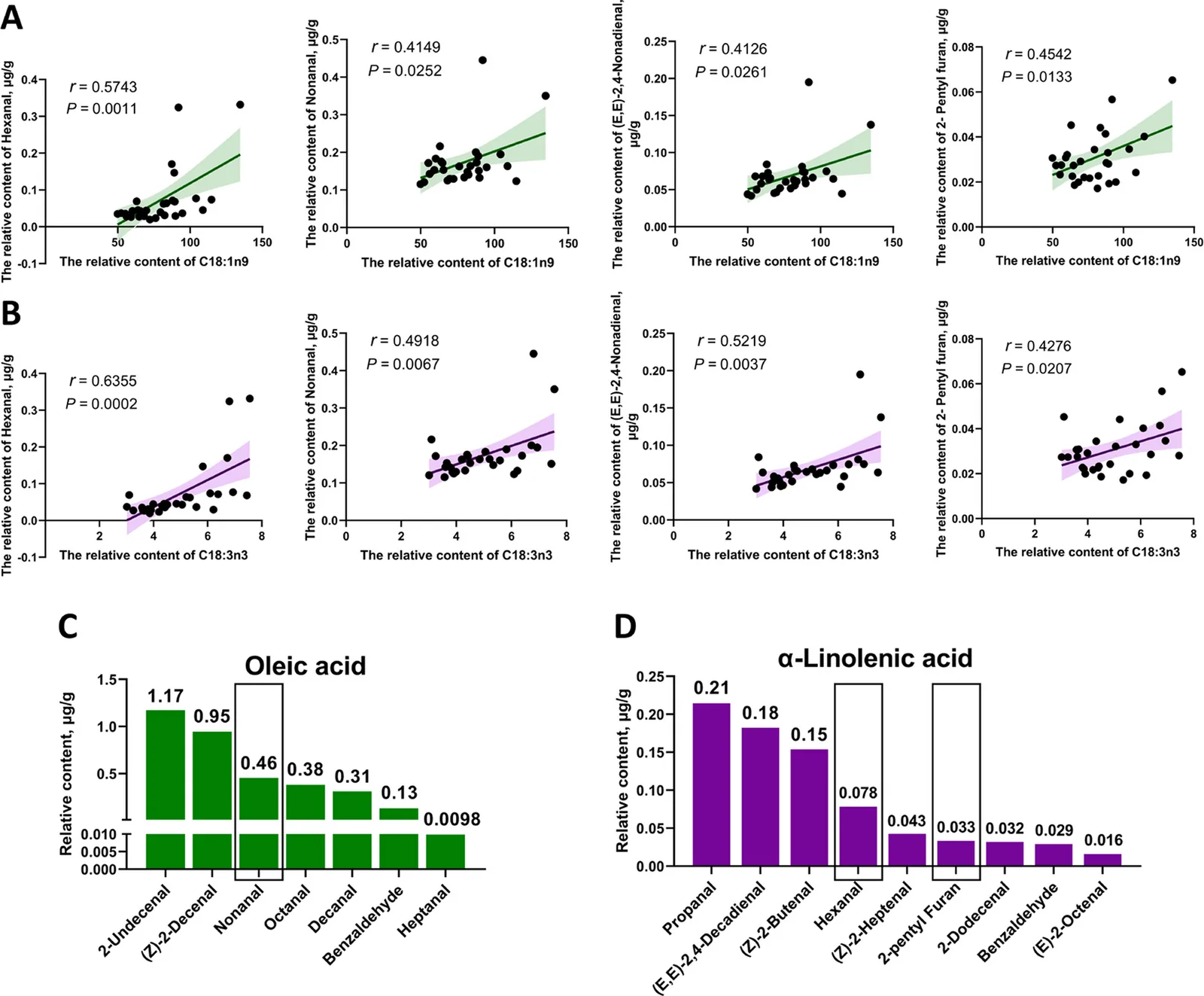
Thermal-oxidative generation of characteristic aldehydes from unsaturated fatty acids. **A** Correlation between oleic acid and the characteristic flavor compounds. **B** Correlation between α-linolenic acid and the characteristic flavor compounds. **C** In vitro validation of oleic acid as a flavor precursor. Aldehyde VFCs produced via thermal oxidation of oleic acid under simulated cooking conditions. **D** Aldehyde VFCs produced via thermal oxidation of α-linolenic acid under simulated cooking conditions

Consistent with predictions from the WGCNA, both oleic acid and α-linolenic acid exhibited significant positive correlations with all four characteristic MS pork flavor compounds (*P* < 0.05; Fig. 4A and B). To validate their role as precursors, oleic acid and α-linolenic acid standards were subjected to thermal treatment under conditions simulating pork cooking. Analysis of VFCs revealed that heating oleic acid generated nonanal (Fig. 4C), while heating α-linolenic acid yielded hexanal and 2‑pentylfuran (Fig. 4D).

### Transcriptomic insights into the regulation of flavor precursor biosynthesis

**Fig. 5:**
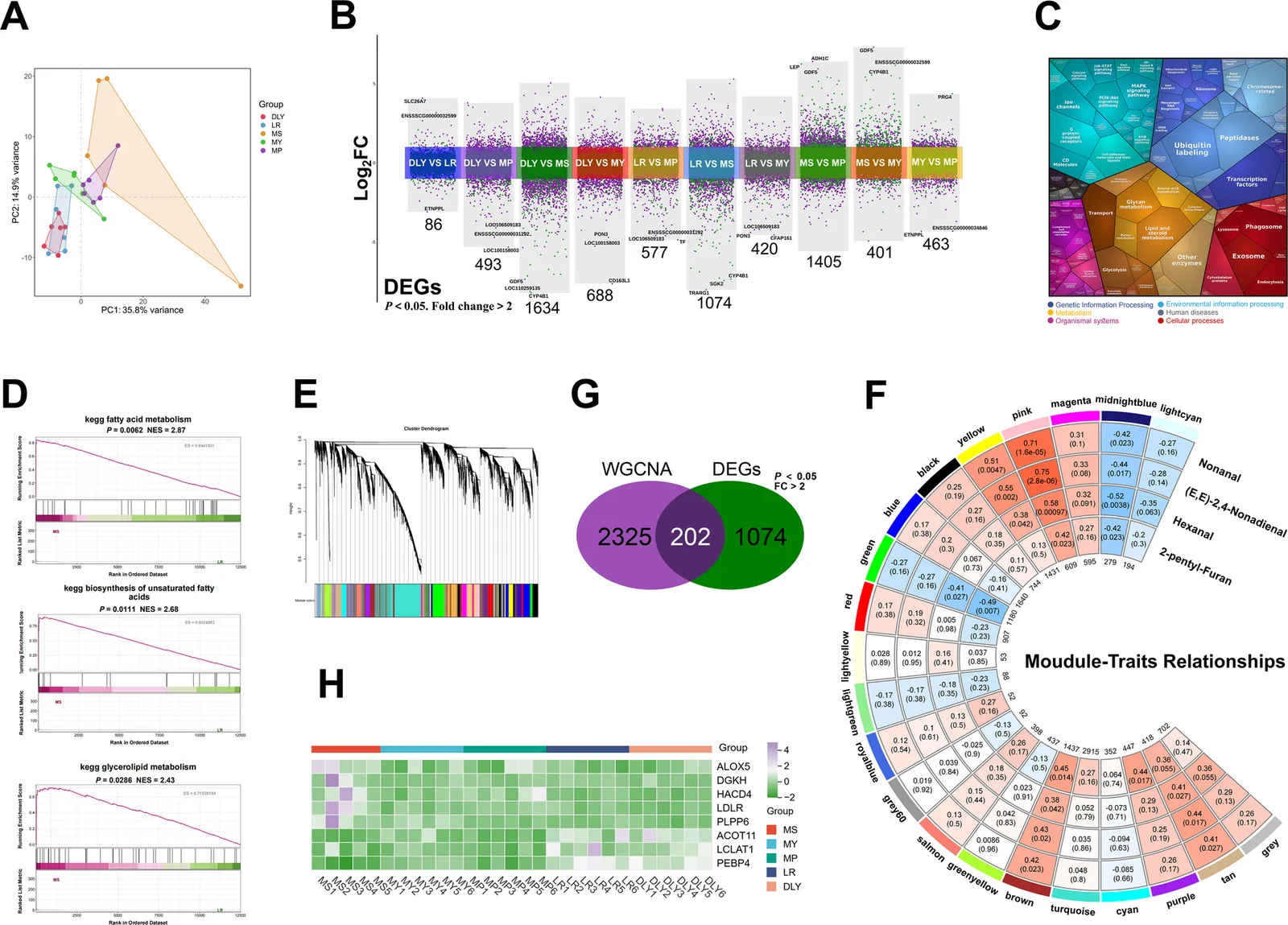
Transcriptomic insights into the regulation of flavor precursor biosynthesis. **A** PCA of the transcriptomic profiles. **B** Pairwise differential expression analysis.​ Volcano plots summarizing the number of differentially expressed genes (DEGs) for all pairwise comparisons between the five breeds. **C** KEGG pathway enrichment analysis of DEGs (MS vs. LR). **D** Gene Set Enrichment Analysis of the transcriptome (MS vs. LR) using the KEGG database. **E** Clustering dendrogram of genes from transcriptome-based WGCNA. Each branch tip represents a single metabolite, and each color band below the dendrogram denotes a distinct module. **F** Module–trait correlation heatmap. Each cell shows the correlation coefficient and significance. **G** Identification of high-confidence candidate regulators via intersection of WGCNA-derived and differentially expressed genes. **H** Functional annotation screening identified eight genes directly associated with lipid metabolism from 202 candidate regulators uncovered by WGCNA and differential expression analysis

To explore the transcriptional regulatory network underlying the formation of VFCs, transcriptome sequencing of the *longissimus thoracis* muscle was conducted. PCA of the transcriptomic data reflected genetic relatedness among breeds, with MS pigs clustering closer to their crossbreeds (MY and MP) and more distantly from the exotic breeds (LR and DLY) (Fig. 5A). Pairwise differential expression analysis identified varying numbers of differentially expressed genes (DEGs) between groups, ranging from 86 to 1,634 (Fig. 5B). Focusing on the most phenotypically divergent breed pair, we performed KEGG pathway enrichment analysis on DEGs between MS and LR pigs, which revealed significant enrichment in the "Lipid and steroid metabolism" pathway (Fig. 5C), consistent with the observed differences in fatty acid composition. Gene Set Enrichment Analysis (GSEA) using the KEGG database further corroborated this finding, showing significant enrichment for pathways including "fatty acid metabolism", "biosynthesis of unsaturated fatty acids", and "glycerolipid metabolism" (Fig. 5D). Similar lipid-related pathways were also enriched in GSEA performed with the Reactome and Wikipathways databases (Fig. S3A and B).

To systematically identify gene co-expression modules associated with the characteristic flavor compounds of MS pork, we performed WGCNA, with the concentrations of the four key VFCs set as phenotypic traits. This analysis identified 21 distinct gene co-expression modules (Fig. 5E; module sizes in Fig. S3C). Module–trait correlation analysis uncovered that nonanal was markedly correlated with the pink, yellow, tan, brown and midnightblue modules; (E,E)-2,4‑nonadienal with the pink, yellow, tan, brown and midnightblue modules; hexanal with the pink, yellow, tan, brown, midnightblue and green modules; and 2‑pentylfuran with the pink, brown, midnightblue, green and purple modules (Fig. 5F). Notably, the pink, brown, and midnightblue modules were commonly associated with all four flavor compounds and collectively harbored 2,325 genes. Intersection of this set with the 1,074 DEGs from the MS vs. LR comparison yielded 202 overlapping genes (Fig. 5G, Table S6), underscoring their critical role as potential key regulators. From these candidates, eight genes (*ACOT11*, *ALOX5*, *DGKH*, *HACD4*, *LCLAT1*, *LDLR*, *PEBP4*, and *PLPP6*) directly implicated in lipid metabolism were identified, with hypothesized functions in modulating precursor lipid deposition to influence flavor compound abundance (Fig. 5H).

### *ALOX5* is involved in promoting aldehyde formation in vivo

**Fig. 6:**
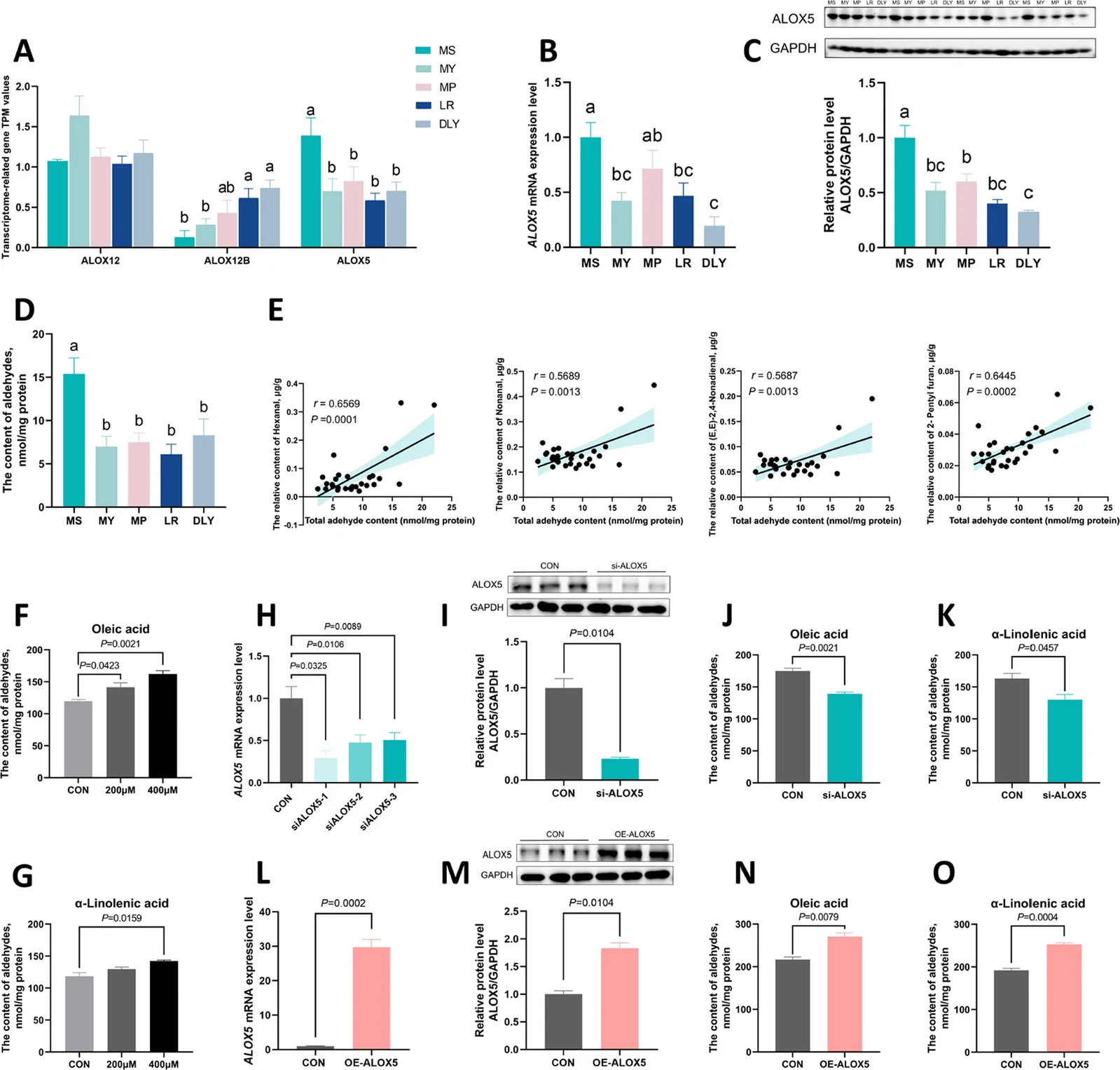
*ALOX5* is involved in promoting aldehyde formation in vivo. **A** Transcriptomic expression profile of the lipoxygenase (LOX) gene family across five pig breeds. **B** Validation of elevated *ALOX5* mRNA levels in Meishan pigs by qPCR. **C** Western blot confirmation of higher ALOX5 protein abundance. **D** Total aldehyde content in unheated muscle. **E** Correlation between total aldehydes and the characteristic flavor compounds. **F** Effect of oleic acid treatment on intracellular aldehyde generation in C2C12 myoblasts. **G** Effect of α-linolenic acid treatment on intracellular aldehyde generation in C2C12 myoblasts. **H** Efficiency of *ALOX5* knockdown by siRNA validated by qPCR. **I** Validation of *ALOX5* knockdown at the protein level by Western blot. **J** Effect of *ALOX5* knockdown on intracellular aldehyde levels in the presence of oleic acid. **K** Effect of *ALOX5* knockdown on intracellular aldehyde levels in the presence of α-linolenic acid. **L** and **M** Validation of *ALOX5* overexpression in C2C12 cells. **N** and **O** Intracellular aldehyde levels upon *ALOX5* overexpression with fatty acid treatment. Data are mean ± SEM (*n* = 5 or 6). Different letters denote significant differences (*P* < 0.05) among groups

Of the 202 overlapping genes, *ALOX5* was prioritized as a candidate regulator. Notably, it was the only lipoxygenase gene that displayed the highest expression in Meishan pigs among all lipid-oxidizing enzymes detected in the transcriptome (*P* < 0.05; Fig. 6A). The elevated expression of ALOX5 in Meishan pigs was validated at both the transcriptional (*P* < 0.01) and translational levels (*P* < 0.05; Fig. 6B and C). Furthermore, *ALOX5* transcript abundance exhibited a markedly positive correlation with the abundance of characteristic flavor substances (*P* < 0.01; Fig. S4A). The total aldehyde content in unheated pork was measured, and the results showed that Meishan pigs had significantly higher intramuscular total aldehyde levels than the other four breed groups (*P* < 0.01; Fig. 6D). Furthermore, these total aldehyde levels showed strong positive correlations with all four characteristic flavor compounds (*P* < 0.01; Fig. 6E).

To validate ALOX5 in a controlled system, studies were conducted in mouse myoblast C2C12 cells, considering the high sequence and functional conservation (91.54%) of this enzyme between pigs and mice (Fig. S4B). Treatment with oleic acid or α-linolenic acid at 400 μmol/L, a concentration that did not affect cell viability (Fig. S4D), significantly increased intracellular aldehyde content (*P* < 0.05; Fig. 6F and G). The lack of change in ALOX5 expression following fatty acid treatment raises the possibility that its elevated enzymatic activity, rather than its mRNA or protein level (Fig. S4C), contributes to the observed phenotype. To further establish the enzymatic role of ALOX5, its expression was knocked down in C2C12 cells (*P* < 0.05; Fig. 6H and I). This knockdown significantly attenuated the aldehyde production induced by either 400 μmol/L oleic acid or 400 μmol/L α-linolenic acid treatment (*P* < 0.05; Fig. 6J and K). Conversely, overexpression of ALOX5 (*P* < 0.05; Fig. 6L and M) enhanced (*P* < 0.01) the aldehyde-generating capacity of the cells in response to the same fatty acid substrates (400 μmol/L) (Fig. 6N and O). Taken together, these findings demonstrate that the breed-specific elevation of ALOX5 in Meishan pigs functions as a key enzymatic driver for the enhanced generation of its characteristic aldehyde flavor compounds.

## Discussion

This study compared volatile profiles across five pig breeds; integrated multi-omics analyses then identified the key flavor compounds, precursors, and transcriptional regulators defining Meishan pork. The results demonstrate that Meishan pork exhibits a unique volatile fingerprint, characterized by significantly elevated levels of hexanal, nonanal, (E,E)-2,4-nonadienal, and 2-pentylfuran. Given their high odor activity, this specific combination is thus proposed as the molecular basis that would be expected to produce its distinctive flavor attributes. Through WGCNA and experimental validation, oleic acid and α‑linolenic acid were established as critical lipid precursors for these flavor compounds, capable of generating them upon thermal oxidation. Furthermore, the elevated expression of *ALOX5* in Meishan pigs was shown to enhance aldehyde formation in vivo prior to cooking. These findings not only provide mechanistic insights into the molecular foundation behind the flavor quality of Meishan pork but also offer potential targets for genetic or nutritional strategies aimed at enhancing flavor attributes in commercial pork production.

Meishan pork is characterized by a comprehensive enrichment of aldehydes, with hexanal, nonanal, (E,E)-2,4-nonadienal, and 2-pentylfuran defined as its characteristic flavor compounds. This is consistent with previous observations that different pig breeds develop distinct volatile flavor fingerprints due to variations in genetic background, diet, and rearing practices [8, 29], and partially aligns with Wu et al. [7], who identified hexanal as a discriminative odorant in Chinese indigenous pigs, along with other aldehydes structurally similar to our signature compounds. Notably, these four compounds are among the 15 key odorants (OAV > 1) identified in pork within a comparative flavor study across different meat types [30]. Thus, the flavor of Meishan pork appears to represent a selective intensification of the core odorant spectrum characteristic of pork. The PCA plots showed considerable overlap among the five breeds, as PCA captures overall variation across all volatiles, most of which are shared among pork breeds. The four signature compounds, however, were identified by univariate comparisons (ANOVA). Thus, the lack of PCA separation reflects selective enrichment of a few key odorants rather than a lack of breed‑specific differences. Hexanal is described as having green, grassy notes; nonanal contributes citrus, orange peel, and fatty notes; (E,E)-2,4-nonadienal generates fatty, waxy, green and fruity aromas; and 2-pentylfuran presents a complex profile including fishy, roasted, and beany notes. Prior research has documented a negative correlation between (E,E)-2,4-nonadienal and soy sauce, caramel, and spicy flavor profiles of braised pork [31], suggesting that it may primarily contribute to a “fatty” or “rich” sensory impression. These four compounds represent key aroma components associated with the characteristic flavors of pork and beef broths [32–34], and some are commonly used in flavor formulations [35]. Moreover, fermented feed supplementation has been reported to significantly increase the contents of (E,E)-2,4-nonadienal, nonanal, and other aldehydes in pork [36, 37], suggesting that these flavor compounds are also amenable to nutritional regulation. Their distinct odor profiles, either individually or through interactive effects within the complex volatile mixture, differentiate Meishan pork from its crossbred and commercial counterparts, and likely underlie its unique flavor characteristics and consumer preference.

In the present study, Meishan pigs and their crossbred offspring exhibited higher IMF content and elevated levels of a broader range of fatty acids compared with the commercial breeds, consistent with previous reports [15]. Notably, although Meishan pigs had the lowest body weight, they displayed the highest IMF content and flavor compound levels, indicating that the breed‑specific genetic program governing lipid deposition overrides the potential negative influence of lower body weight on flavor formation [38]. Particularly noteworthy is the significantly lower N6/N3 polyunsaturated fatty acid (PUFA) ratio in Meishan pigs, a metabolic signature that confers potential immune and metabolic benefits [39, 40]. This advantageous fatty acid composition, including the lower N6/N3 ratio, along with higher IMF, was preserved in the crossbreds and distinguished them from the LR and DLY breeds.

Our results confirm that oleic acid (C18:1n9) generates nonanal upon thermal oxidation, while α‑linolenic acid (C18:3n3) serves as a direct precursor of hexanal and 2‑pentylfuran. These findings align with the established view that unsaturated fatty acids serve as crucial substrates in meat flavor formation [13, 29]. Specifically, C18:1, C18:2, C18:3, and C20:4 are recognized as key precursors of lipid‑derived volatiles, as unsaturated fatty acids oxidize far more readily than saturated ones, promoting the formation of aroma‑active aldehydes and other VFCs [41]. Among these, oleic acid has been previously reported to generate nonanal [16], and our results confirm this conversion. In contrast, hexanal has traditionally been attributed to linoleic acid (C18:2n6) oxidation [9]; however, our study provides the first direct evidence that α‑linolenic acid can also yield hexanal, as well as 2‑pentylfuran. Mechanistically, the formation of 2‑pentylfuran from α‑linolenic acid is consistent with the known pathway in which the fatty acid is first oxidatively cleaved to 2‑nonenal, followed by cyclization [42]. Thus, all four signature flavor compounds of Meishan pork originate directly from aldehyde formation or its subsequent modification. Indeed, the elevated lipid pool in IMF‑rich pigs serves as a rich precursor reservoir, accounting for over half of the VFCs generated in cooked pork through lipid oxidation and degradation [9]. Meat aroma development is primarily attributed to phospholipids within the IMF, which contain a high proportion of PUFAs and are considered the major precursors for pork flavor [43]. Supplementation with phospholipids has been shown to enhance meat aroma specifically through the formation of aldehydes [44]. This is consistent with our observation that the high IMF content in Meishan pigs provides abundant unsaturated fatty acid precursors for aldehyde generation. While WGCNA and in vitro heating experiments collectively pinpointed oleic acid and α‑linolenic acid as critical precursors, the complete set of four characteristic flavor compounds could not be fully reconstituted from these two fatty acids alone. This limitation likely reflects the inherent complexity of in vivo flavor formation, which involves synergistic interactions among diverse lipids, proteins, and carbohydrates, as well as a dynamic network of metabolic and enzymatic processes rather than simple thermochemistry. Moreover, although we focused on lipid‑derived volatiles, water‑soluble precursors also play crucial roles in forming meaty and roasted aromas via the Maillard reaction [45], and WGCNA revealed modules co‑enriched in water‑soluble metabolites correlated with our key flavor compounds, suggesting that multiple pathways may collectively shape the complete flavor profile of Meishan pork.

Transcriptomic analysis revealed a marked upregulation of lipid and steroid metabolic pathways, including fatty acid biosynthesis, in Meishan pigs, corresponding to their phenotypic traits of high IMF and elevated unsaturated fatty acids. To further explore the genetic basis of precursor accumulation and flavor formation, WGCNA was employed to associate gene co‑expression modules with the four key flavor compounds [46]. This systems‑level approach linked the flavor phenotype to coordinated transcriptional programs and identified 202 candidate genes overlapping with differentially expressed genes. This set constitutes a putative transcriptional network that may coordinately modulate lipid metabolism, thereby influencing the pool of flavor precursors in Meishan pigs. While previous research on pork flavor has largely centered on the characterization of VFCs and their precursors, with genetic regulatory mechanisms remaining comparatively underexplored, transcriptomic studies in other species have successfully uncovered candidate regulatory genes involved in the biosynthesis of several key VFCs—for example, elucidating the regulation of 1‑octen‑3‑ol formation via the mevalonate pathway in chicken [47] and identifying specific genes (*SLC27A1*) and signaling pathways (PPAR) associated with hexanal content [48].

Following its initial identification based on a known role in lipid oxidation, *ALOX5* was further prioritized due to its uniquely high expression in Meishan pigs and subsequently validated by a strong correlation with the characteristic flavor compounds. Lipoxygenases drive oxidation of polyunsaturated fatty acids including arachidonic, linoleic, and α-linolenic acid. This reaction initiates a cascade yielding hydroperoxides and volatile short-chain carbonyls, including the aldehydes and alcohols central to meat aroma [18, 49, 50]. While *ALOX5* activity is known to generate aldehydes from substrates like linoleic acid [22], our studies specifically demonstrated its critical role in converting oleic acid and α-linolenic acid into key flavor aldehydes. Notably, supplementation with precursor fatty acids did not upregulate *ALOX5* expression in C2C12 cells. This finding suggests that the enhanced production of aldehydes observed in functional assays is likely mediated through an increase in ALOX5 enzymatic activity, rather than through elevated gene expression. Although GC‑MS profiling of specific volatile aldehydes in the C2C12 system was precluded by the limited cell biomass, total aldehyde quantification served as a reliable proxy based on its strong correlation with the four characteristic flavor compounds in muscle tissue. Together, the high basal *ALOX5* expression and the elevated aldehydes in unheated muscle indicate that a portion of the characteristic flavor compounds in Meishan pork is generated in vivo prior to cooking.

**Fig. 7:**
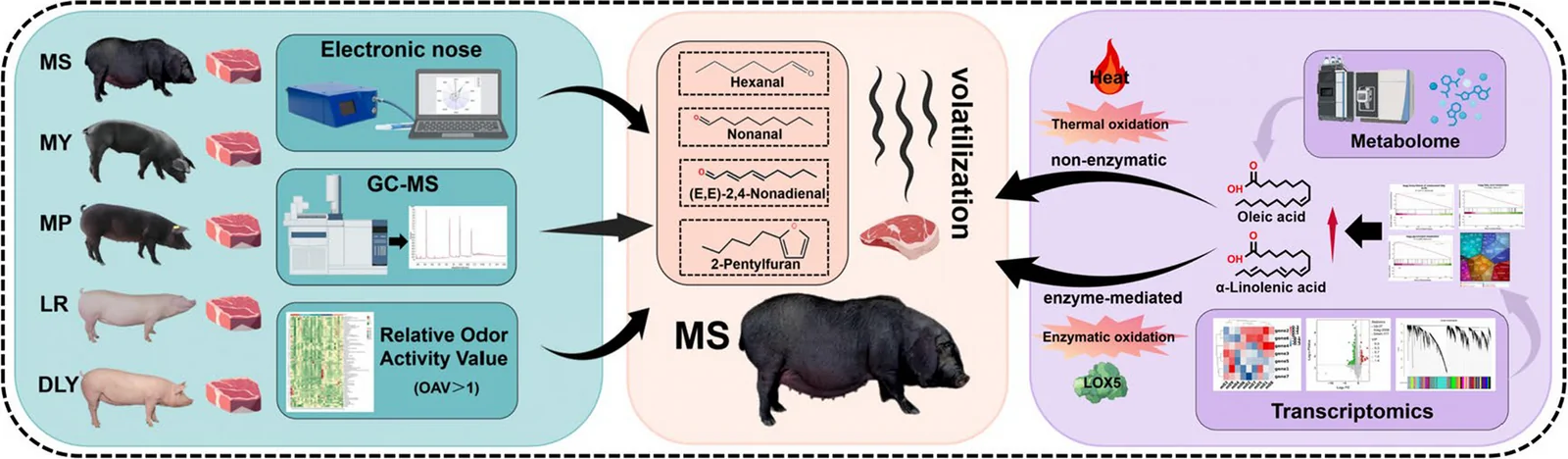
Schematic model illustrating the integrated workflow from identification of characteristic flavor compounds to elucidation of their dual-pathway formation mechanism in Meishan pork

In summary, this study proposes an integrated "gene–metabolism–flavor" model for the characteristic flavor formation in Meishan pork (Fig. 7). The unique genetic background of Meishan pigs drives the specific upregulation of a lipid metabolic network, which promotes intramuscular fat deposition and enriches the pool of unsaturated fatty acid precursors. The constitutively high activity of ALOX5 initiates enzymatic oxidation in vivo, leading to the accumulation of flavor aldehydes prior to cooking. During cooking, the abundant lipid substrates undergo intense thermal oxidation, synergizing with these pre-formed enzymatic products to generate and release the characteristic VFCs in high abundance. This work delivers a mechanistic explanation for the superior flavor of Meishan pork, spanning from molecular regulation to the final sensory trait, and identifies direct targets for molecular breeding strategies aimed at flavor improvement. The interpretations drawn from this work are subject to certain limitations, as phospholipid composition, antioxidant status, and postmortem oxidative processes were not examined and may also contribute to the observed differences [51]. Future research should prioritize two key directions to build upon these findings. First, given the species-specific differences in fatty acid profiles, intramuscular fat deposition, and myofiber composition between pigs and mice [52–54], the function of ALOX5 and its impact on the overall flavor phenotype require validation in more physiologically relevant systems, such as porcine primary myocytes or gene-edited animal models. Second, sensory-guided assays are needed to quantify the impact of the identified flavor compounds on the overall palatability of Meishan pork. This essential step would complete the translation from "chemical fingerprint" to "consumer preference", thereby closing the final loop between molecular discovery and sensory perception.

## Conclusion

This study integrated volatilomics, metabolomics, and transcriptomics to decipher the distinctive flavor of Meishan pork. The four key flavor compounds identified—hexanal, nonanal, (E,E)-2,4-nonadienal, and 2-pentylfuran—derive their elevated abundance from increased levels of the precursor fatty acids, oleic acid and α‑linolenic acid. A breed‑specific transcriptional network promoting lipid deposition underpins this precursor enrichment. Among the identified candidates, *ALOX5* was validated as a gene that contributes to the enzymatic conversion of precursors to flavor aldehydes, and its elevated expression in Meishan pigs is associated with enhanced conversion efficiency, suggesting one of the regulatory pathways from gene to flavor. While this study highlights a potentially important pathway, future work should explore the upstream regulation of *ALOX5* and validate the sensory impact of the identified compounds. Overall, these findings provide a molecular framework for understanding pork flavor and offer potential targets for precision breeding aimed at meat quality improvement.

## Supplementary Information

Additional file 1: Table S1. Ingredients and nutrient contents of the experimental diets. Table S2. Performance description and sensitivity of metal oxide sensors for PEN3 electronic nose. Table S3. Primers used in quantitative real-time PCR analysis. Table S4. Antibodies used for Western blot analysis. Table S5. The key volatile flavor compounds with OAV > 1 in five pig breeds. Table S6. List of 202 candidate key regulatory genes identified by intersecting flavor-associated WGCNA modules with differentially expressed genes. Fig. S1. Statistical comparison of E-nose sensor responses. Fig. S2. Size distribution of the metabolite co-abundance modules identified by WGCNA. Fig. S3. Functional enrichment analysis and gene co-expression module characterization. Fig. S4. Functional characterization and sequence analysis of *ALOX5* in relation to flavor formation.

Additional file 2: Classification and relative contents of volatile flavor compounds across five pig breeds.

Additional file 3: Original Western blot images.

## Data Availability

All relevant data are presented within the article and supplementary materials. Further data are available from the corresponding author on request.

## Abbreviations

ALOX5: Arachidonate 5-lipoxygenase
DEGs: Differentially expressed genes
E-nose: Electronic nose
GC-MS: Gas chromatography-mass spectrometry
GS: Gene significance
GSEA: Gene set enrichment analysis
HS-SPME: Headspace solid-phase microextraction
IMF: Intramuscular fat
KEGG: Kyoto encyclopedia of genes and genomes
LOXs: Lipoxygenases
MM: Module membership
rOAV: Relative odor activity value
TGs: Triglycerides
VFCs: Volatile flavor compounds
WGCNA: Weighted correlation network analysis
