# Supplementary material for: Integrated multi-omics analysis deciphers the molecular mechanisms underlying the characteristic volatile flavor of Meishan pork
Source: J Anim Sci Biotechnol. 2026 Nov 4;17:199. doi: 10.1186/s40104-026-01515-7 (PMC13733252; doi:10.1186/s40104-026-01515-7)

Figgure 6. ALOX5

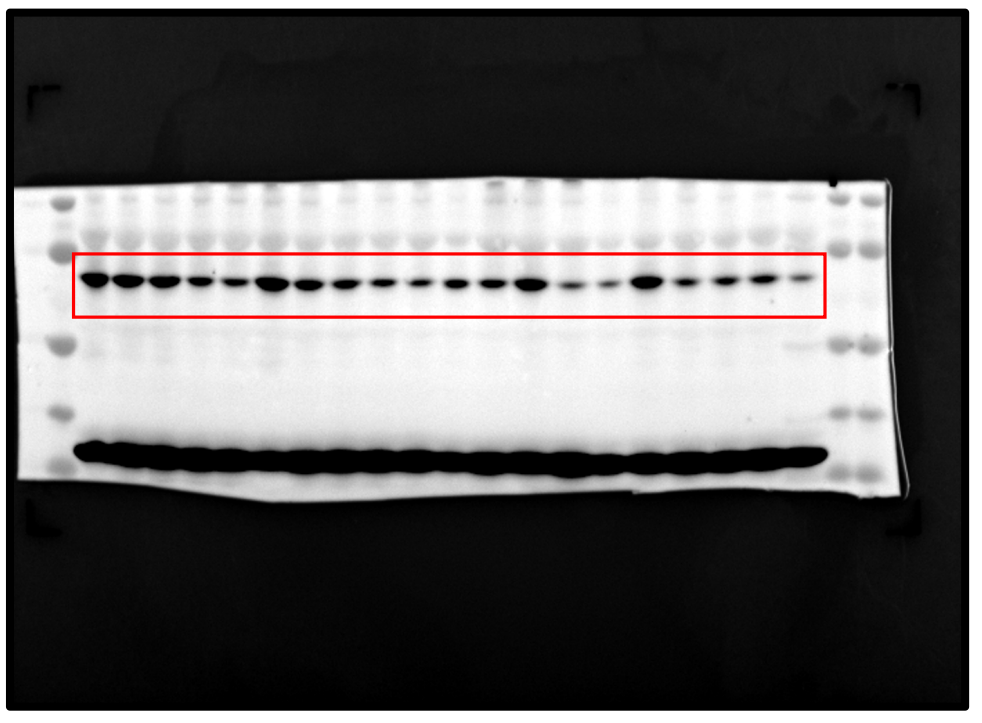

Figgure 6. GAPDH

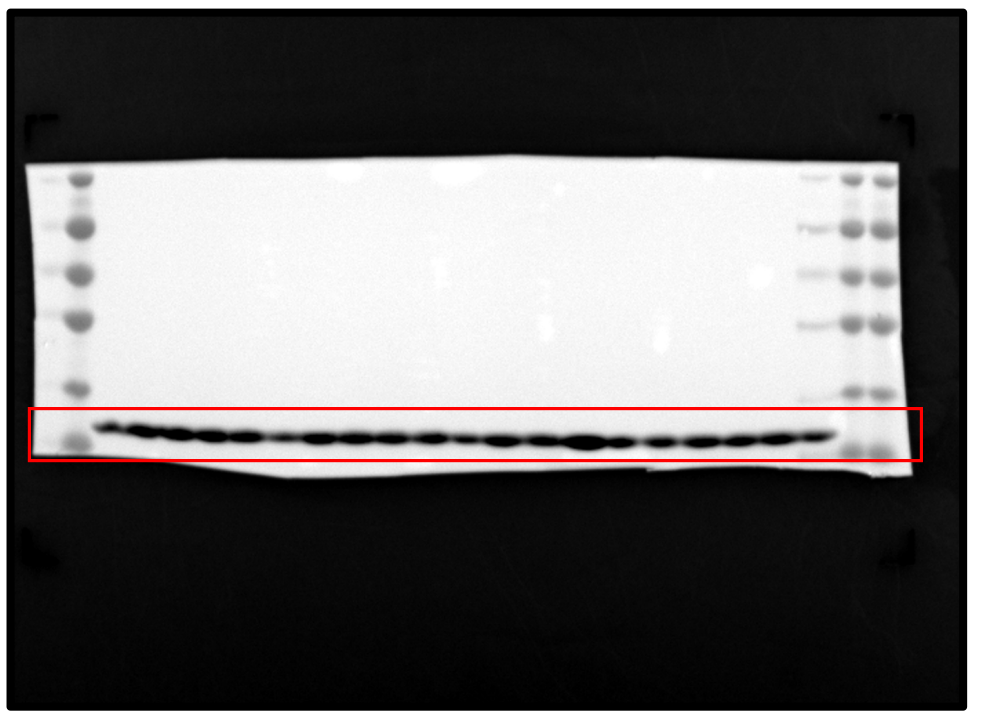

Figgure 6. ALOX5

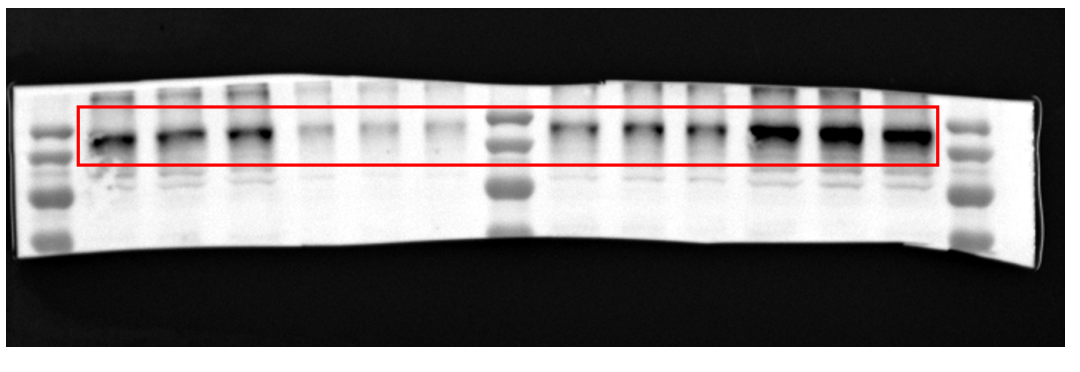

Figgure 6. GAPDH

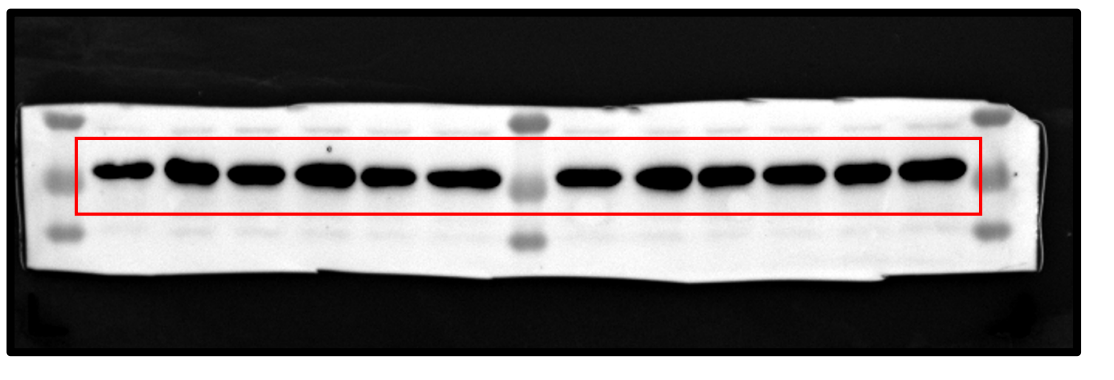

Supplementary figure 4C

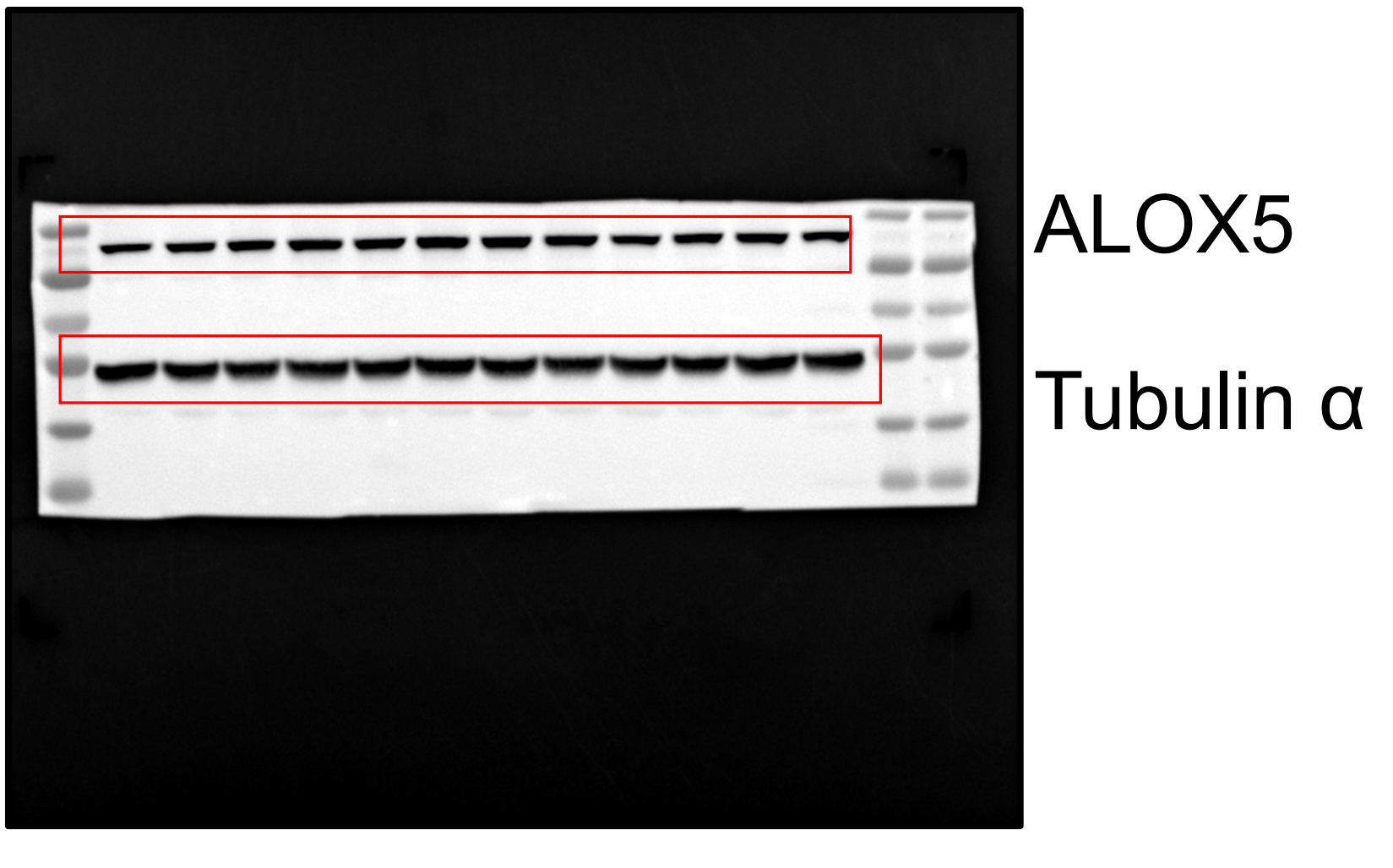

Supplement: Supplementary file 1 [file 40104_2026_1515_MOESM3_ESM.pdf]
