# Supplementary material for: Integrated multi-omics analysis deciphers the molecular mechanisms underlying the characteristic volatile flavor of Meishan pork
Source: J Anim Sci Biotechnol. 2026 Nov 4;17:199. doi: 10.1186/s40104-026-01515-7 (PMC13733252; doi:10.1186/s40104-026-01515-7)
Supplement: Supplementary file 2 [file 40104_2026_1515_MOESM1_ESM.docx]

Table S1. Ingredients and nutrient contents of the experimental diets (DM basis,%).

| **Ingredient** | **%** | **Nutrient level** | **%** |
| --- | --- | --- | --- |
| Dry matter | 88.85 | Crude protein | 17.83 |
| Corn | 71.21 | Ether extract | 3.93 |
| Soybean meal | 16.65 | Calcium | 0.49 |
| Wheat bran | 8.21 | Total phosphorus | 0.45 |
| Wheat middlings | 1.00 | Available phosphorus | 0.22 |
| Premix^1^ | 1.00 | Lysine | 1.10 |
| Limestone | 0.80 | Methionine + Cystine | 0.55 |
| Lysine | 0.61 | Metabolizable energy, MJ•kg^-1^ | 12.38 |
| CaHPO_4_ | 0.50 | Crude fiber | 3.45 |
| Methionine | 0.02 | Neutral detergent fiber | 9.58 |
| Soybean oil | 0.00 | Acid detergent fiber | 3.05 |
| Total | 100.00 | Hemicellulose | 6.53 |
|  | | Insoluble dietary fiber | 15.94 |
|  |  | Soluble dietary fiber | 0.65 |
|  |  | Total dietary fiber | 16.59 |

^1^ The premix provided the following per kg of diets: vitamin A 320 000 IU, vitamin D3 80 000 IU, vitamin E 1 200 mg, vitamin B1 80 mg, vitamin B2 130 mg, niacin 300 mg, pantothenic acid 640 mg, biotin 10 mg, Fe 5 600 mg, Cu 800 mg, Zn 1 600 mg, Mn 2 000 mg.

^2^ In the nutrition level, Calcium, Phosphorus, Lysine, Methionine + cystine and metabolic energy of pigs are calculated values, while other indicators are measured values.

Table S2. Performance description and sensitivity of metal oxide sensors for PEN3 electronic nose.

| **No.** | **Sensor name** | **Performance description** | **Reference Threshold** |
| --- | --- | --- | --- |
| 1 | W1C | Benzene and Aromatic Compounds | Methylbenzene, 10 ppm |
| 2 | W5S | Nitrogen Oxides | Nitrogen dioxide, 1 ppm |
| 3 | W3C | Ammonia & Aromatics | Benzene, 10 ppm |
| 4 | W6S | Hydrogenated Compounds | Hydrogen, 100 ppm |
| 5 | W5C | Short-chain Alkanes & Aromatics | Propane, 1 ppm |
| 6 | W1S | Methane | Methane, 100 ppm |
| 7 | W1W | Sulfur Compounds | Hydrogen sulfide, 1 ppm |
| 8 | W2S | Alcohols & Aldehydes & Ketones | Carbon monoxide, 100 ppm |
| 9 | W2W | Aromatics & Organic Sulfur Compounds | Hydrogen sulfide, 1 ppm |
| 10 | W3S | Long-chain Alkanes | Methane, 100 ppm |

Table S3. Primers used in quantitative real-time PCR analysis.

| **Gene** | **Forward** | **Reverse** |
| --- | --- | --- |
| Pig ALOX5 | TGAACTTCGGCCAGTACGAC | AGCTCGTTGTCCTGGAACTG |
| Pig PPIA | GTCTCCTTCGAGCTGTTTGC | GTCACCACCCTGGCACATAA |
| Mouse ALOX5 | TGACAGGGTCAAGAAGTTGGT | GAGCCAATGAGGCTGAGGTAG |
| Mouse PPIA | GAGCTGTTTGCAGACAAAGTTC | CCCTGGCACATGAATCCTGG |

Table S4. Antibodies used for Western blot analysis.

| **Antibody** | **Company** | **Item No.** | **Dilution ratio** |
| --- | --- | --- | --- |
| ALOX5 | ZEN-BIOSCIENCE | R382216 | 1:1000 |
| GAPDH | ABclonal Technology | AC054 | 1:10000 |

Table S5. The key volatile flavor compounds with OAV > 1 in five pig breeds.

| **Volatile compound** | **Threshold**  **(ug/kg)** | **Odor** | **OAV value** | | | | | **SEM** | ***P* valve** |
| --- | --- | --- | --- | --- | --- | --- | --- | --- | --- |
|  |  |  | **MS** | **MY** | **MP** | **LR** | **DLY** |  |  |
| 2-methoxy-3-(2-methylpropyl)-Pyrazine | 0.00 | green bell pepper, pea, galbanum | 3957080.39 ± 893136.31c | 4799728.04 ± 872839.98abc | 4341336.77 ± 840618.51bc | 5349152.26 ± 785396.46ab | 5598676.72 ± 1086235.51a | 191973.63 | 0.0306 |
| dihydro-2-methyl-3(2H)-Furanone | 0.01 | sweet, solvent, bread, buttery, nutty | 1440715.43 ± 171312.47 | 1658844.31 ± 233269.96 | 1594471.61 ± 182083.04 | 1513370.03 ± 178802.78 | 1608683.72 ± 211137.86 | 36815.03 | 0.4073 |
| 2,2,6-trimethyl-Cyclohexanone | 0.10 | pungent, thujone, labdanum, honey, cistus | 4955.26 ± 1254.53 | 5118.46 ± 1043.56 | 4539.32 ± 994.17 | 5063.15 ± 835.24 | 5584.18 ± 678.94 | 178.34 | 0.4826 |
| Dimethyl triSulfur compounds | 0.01 | sulfury, cooked onion, savory, meaty | 4003.52 ± 1196.18ab | 2851.55 ± 943.89bc | 2431.35 ± 449.37c | 3854.94 ± 1204.51abc | 4891.54 ± 1842.04a | 267.66 | 0.0147 |
| 4-(2,6,6-Trimethylcyclohexa-1,3-dienyl)but-3-en-2-one | 0.09 | - | 1016.89 ± 324.99bc | 1012.01 ± 277.77c | 828.85 ± 228.70c | 1339.29 ± 214.57ab | 1487.47 ± 244.58a | 63.91 | 0.0011 |
| 3-Octanone | 1.30 | fresh, herbal, lavender, sweet, mushroom | 448.79 ± 210.39b | 445.41 ± 129.23b | 312.08 ± 60.14b | 797.05 ± 107.44a | 831.30 ± 506.14a | 59.78 | 0.0057 |
| 2-Hexenal, (E)- | 3.10 | green, grassy | 402.13 ± 78.10 | 499.82 ± 71.43 | 454.20 ± 32.69 | 465.51 ± 41.31 | 497.85 ± 65.60 | 12.13 | 0.0777 |
| alpha-Irone | 2.00 | orris, floral, berry, violet, woody, powdery | 372.10 ± 120.24bc | 371.90 ± 96.79bc | 318.44 ± 79.57c | 441.16 ± 82.74ab | 522.48 ± 70.27a | 20.55 | 0.0072 |
| Octanal | 0.70 | lemon, citrus, greenl grass | 586.79 ± 162.78 | 408.46 ± 113.98 | 403.14 ± 94.02 | 489.12 ± 108.60 | 516.91 ± 84.62 | 23.32 | 0.0654 |
| beta-Ionone | 0.01 | floral, woody, sweet, fruity, berry, tropical, beeswax | 226.20 ± 24.90 | 271.06 ± 68.36 | 253.93 ± 63.15 | 273.26 ± 83.15 | 331.51 ± 113.33 | 14.82 | 0.2645 |
| (E,E)-2,4-Nonadienal | 0.16 | fatty, melon, waxy, green, violet, leafy, cucumber, tropical, fruity, chicken | 649.78 ± 386.53a | 429.82 ± 26.65b | 418.20 ± 39.10b | 309.67 ± 49.43b | 372.18 ± 81.6b | 35.16 | 0.0306 |
| Hexanoic acid ethyl ester | 5.00 | apple, pear, fruity | 236.6 0± 92.85bc | 183.22 ± 33.42c | 144.08 ± 24.20c | 337.27 ± 63.24ab | 418.84 ± 157.90a | 24.64 | 0.0001 |
| 2,4-Undecadienal | 0.01 | green, buttery, spicy, baked, fruity, fatty, aldehydic, chicken | 495.60 ± 134.91 | 480.82 ± 51.02 | 418.91 ± 107.67 | 413.08 ± 54.87 | 385.59 ± 96.26 | 17.73 | 0.2452 |
| 2-Octen-1-ol | 50.00 | green, vegetable | 259.93 ± 38.97 | 264.41 ± 38.74 | 239.74 ± 32.31 | 272.96 ± 31.97 | 297.98 ± 48.68 | 7.57 | 0.1620 |
| Decanal | 0.10 | sweet, aldehydic, waxy, orange peel, citrus, floral | 232.90 ± 56.07a | 200.79 ± 57.94ab | 180.87 ± 25.17bc | 273.76 ± 43.23c | 295.44 ± 39.47c | 11.38 | 0.0009 |
| (E)-2-Octenal | 3.00 | fresh, cucumber, fatty, green, herbal, banana, waxy, leafy | 215.41 ± 31.58 | 217.52 ± 33.39 | 198.13 ± 25.09 | 230.42 ± 25.44 | 251.16 ± 40.84 | 6.43 | 0.0893 |
| p-Cresol | 0.24 | phenol, narcissus, animalic, mimosa | 211.47 ± 30.36 | 220.28 ± 28.64 | 204.23 ± 21.63 | 225.11 ± 28.83 | 245.10 ± 44.50 | 6.07 | 0.2578 |
| 2-methoxy-Phenol | 1.60 | nutty | 133.63 ± 39.06 | 135.49 ± 37.88 | 125.98 ± 23.75 | 144.50 ± 14.54 | 170.61 ± 30.85 | 5.98 | 0.1361 |
| Nonanal | 1.00 | aldehyde, citrus, orange peel | 256.77 ± 135.82a | 167.58 ± 13.76b | 165.42 ± 23.61b | 134.21 ± 20.58b | 156.02 ± 32.00b | 12.71 | 0.0279 |
| Dodecanenitrile | 0.09 | citrus, orange, peel, metallic, spicy | 134.81 ± 45.63 | 101.43 ± 38.40 | 88.07 ± 36.40 | 169.59 ± 93.15 | 145.49 ± 31.03 | 10.92 | 0.0955 |
| Linalool | 6.00 | floral, green | 94.78 ± 23.63 | 77.26 ± 13.36 | 71.29 ± 10.24 | 83.32 ± 12.40 | 92.21 ± 16.32 | 3.13 | 0.0865 |
| 3-methyl-Indole | 0.41 | animalic, fecal, indole, civet | 60.96 ± 60.13 | 38.12 ± 15.96 | 28.14 ± 18.03 | 23.47 ± 22.99 | 34.45 ± 20.91 | 5.73 | 0.3334 |
| (Z)-2-Cyclopenten-1-one, 3-methyl-2-(2-pentenyl) | 0.26 | woody, herbal, floral, spicy, jasmin, celery | 36.81 ± 14.69 | 35.91 ± 15.31 | 29.58 ± 7.95 | 40.96 ± 11.70 | 46.82 ± 20.55 | 2.75 | 0.3613 |
| (Z)-2(3H)-Furanone, dihydro-5-(2-octenyl) | 5.40 | sweet, fatty, waxy, dairy, creamy, fruity | 29.50 ± 12.90ab | 29.15 ± 5.90b | 25.23 ± 9.33b | 41.24 ± 12.34a | 41.90 ± 8.94a | 2.17 | 0.0240 |
| 4-(1-methylethenyl)-1-Cyclohexene-1-carboxAldehyde | 30.00 | fresh, green, herbal, grassy, sweet, minty, cumin | 26.90 ± 5.67bc | 26.87 ± 5.43bc | 25.61 ± 3.54c | 32.00 ± 4.13ab | 33.34 ± 5.87a | 1.04 | 0.0450 |
| Dodecanoic acid methyl ester | 3.50 | waxy, soapy, creamy, coconut, mushroom | 27.78 ± 7.32 | 27.82 ± 8.62 | 26.05 ± 7.04 | 28.15 ± 8.43 | 34.87 ± 6.30 | 1.43 | 0.3334 |
| 1-(4,5-dihydro-2-thiazolyl)-Ethanone | 1.00 | corn, chip, taco, potato, toasted, bread, nutty | 21.10 ± 18.95 | 36.26 ± 20.42 | 28.45 ± 15.60 | 24.27 ± 25.25 | 11.19 ± 9.28 | 3.58 | 0.2433 |
| 5-methyl-2(5H)-Furanone | 200.00 | - | 15.87 ± 11.22 | 21.19 ± 9.26 | 22.19 ± 2.16 | 24.28 ± 9.99 | 19.5 ± 12.3 | 1.73 | 0.6745 |
| E-2-Undecenal | 0.78 | fresh, fruity, citrus, orange, peel | 29.36 ± 9.56 | 27.00 ± 2.48 | 23.20 ± 8.06 | 28.37 ± 6.33 | 29.29 ± 5.64 | 1.23 | 0.5070 |
| 5-heptyldihydro-2(3H)-Furanone | 2.10 | fruity, peach, creamy, fatty, lactonic, apricot, ketonic, coconut | 16.86 ± 7.73ab | 12.87 ± 3.28b | 12.41 ± 4.98b | 22.65 ± 7.73a | 20.82 ± 3.26a | 1.25 | 0.0131 |
| Heptanoic acid ethyl ester | 2.00 | fruity, pineapple, cognac, rummy, wine | 18.79 ± 2.61bc | 18.05 ± 2.75c | 16.74 ± 3.83c | 22.73 ± 3.95b | 27.54 ± 2.84a | 0.94 | ＜0.0001 |
| 2,4-Di-tert-butylphenol | 500.00 | phenol | 11.89 ± 4.76b | 12.49 ± 3.83b | 9.95 ± 2.04b | 19.53 ± 8.60a | 19.99 ± 6.59a | 1.26 | 0.0130 |
| 5-methyl-2-(1-methylethyl)-Cyclohexanone | 4.70 | minty | 15.24 ± 5.22 | 13.97 ± 6.29 | 12.79 ± 3.42 | 14.74 ± 3.76 | 15.80 ± 5.58 | 0.88 | 0.8542 |
| 1,4-diethyl-Benzene | 2.10 | - | 12.02 ± 2.29ab | 9.79 ± 1.49bc | 7.07 ± 0.49c | 15.09 ± 7.70a | 13.77 ± 1.88ab | 0.85 | 0.0098 |
| Methional | 0.20 | musty, potato, tomato, earthy, vegetable, creamy | 7.00 ± 5.48b | 19.46 ± 10.61a | 13.14 ± 9.91ab | 6.73 ± 2.86b | 19.60 ± 11.14a | 1.84 | 0.0377 |
| 1-Octen-3-ol | 1.00 | fatty, fruity, grassy, mushroom, perfumy, sweet | 20.21 ± 5.29 | 12.09 ± 3.92 | 22.35 ± 19.36 | 10.30 ± 3.12 | 12.96 ± 1.30 | 1.85 | 0.1475 |
| gamma-Dodecalactone | 0.43 | fatty, peachy, musky | 7.19 ± 2.28b | 7.50 ± 1.38b | 6.46 ± 1.54b | 10.62 ± 3.75a | 10.39 ± 1.68a | 0.51 | 0.0104 |
| Propanoic acid pentyl ester | 85.00 | sweet, fruity, apricot, pineapple, tropical, banana | 5.29 ± 2.46 | 3.90 ± 2.53 | 2.78 ± 1.41 | 4.04 ± 1.38 | 6.98 ± 4.20 | 0.53 | 0.0957 |
| alpha-Ionone | 3.78 | sweet, woody, floral, violet, orris, tropical, fruity | 7.45 ± 1.91bc | 7.43 ± 1.44bc | 6.49 ± 1.62c | 8.80 ± 1.86ab | 10.57 ± 1.66a | 0.40 | 0.0037 |
| Diallyl Sulfur compounds | 100.00 | sulfury, onion, garlic, horseradish, metallic | 8.88 ± 0.81c | 9.08 ± 1.01bc | 8.15 ± 0.81c | 10.30 ± 0.73a | 10.02 ± 0.65ab | 0.20 | 0.0008 |
| 5-butyldihydro-2(3H)-Furanone | 17.90 | sweet, coconut, waxy, creamy, tonka, dairy, fatty | 13.58 ± 4.73 | 9.56 ± 1.47 | 9.10 ± 2.45 | 13.03 ± 6.75 | 11.21 ± 1.35 | 0.75 | 0.2381 |
| delta-Dodecalactone | 7.60 | peachy, oily, creamy, soapy | 6.38 ± 2.84ab | 6.43 ± 1.49b | 5.49 ± 1.99b | 9.17 ± 2.82a | 9.14 ± 1.87a | 0.48 | 0.0226 |
| 4-Nonanone | 8.20 | - | 6.72 ± 1.83 | 6.77 ± 1.42 | 6.26 ± 0.88 | 7.08 ± 0.75 | 8.49 ± 1.67 | 0.27 | 0.0837 |
| (E)-2-Decenal | 5.00 | waxy, fatty, earthy, green, cilantro, mushroom, aldehydic, fried, chicken, fatty, tallow | 10.51 ± 4.78 | 7.81 ± 1.95 | 7.80 ± 2.08 | 8.90 ± 3.94 | 8.48 ± 1.10 | 0.55 | 0.5776 |
| 1-Octanol | 22.00 | intense citrus, rose | 9.32 ± 2.52 | 8.74 ± 1.14 | 8.67 ± 1.06 | 8.32 ± 2.14 | 9.00 ± 1.45 | 0.30 | 0.8985 |
| 6-Methyl-3,5-heptadiene-2-one | 100.00 | - | 5.08 ± 1.37 | 5.01 ± 1.02 | 4.69 ± 0.66 | 5.45 ± 0.67 | 6.39 ± 1.21 | 0.21 | 0.0676 |
| 2-Decanol | 25.00 | anisic, coconut | 4.09 ± 1.84 | 5.65 ± 2.84 | 6.53 ± 1.14 | 4.94 ± 1.19 | 5.52 ± 0.95 | 0.33 | 0.2356 |
| nitro-Cyclohexane | 150.00 | - | 5.48 ± 1.42 | 5.28 ± 1.45 | 5.01 ±0 .81 | 5.60 ± 0.50 | 6.08 ± 1.17 | 0.20 | 0.5633 |
| (Z)-2-Decenal | 50.00 | tallow | 5.30 ± 1.43abc | 4.74 ± 0.73bc | 4.29 ± 0.54c | 5.99 ± 0.90a | 5.78 ± 1.30ab | 0.21 | 0.0417 |
| Hexanal | 5.00 | aldehyde, grassy, green, leafy, vinegar | 38.59 ± 26.04a | 10.05 ± 2.71b | 13.48 ± 8.63b | 5.88 ± 1.25b | 7.84 ± 3.34b | 2.92 | 0.0007 |
| (E)-3,7-dimethyl-2,6-Octadienal | 28.00 | citrus, lemon | 2.71 ± 0.67 | 3.67 ± 0.68 | 3.68 ± 0.60 | 3.05 ± 0.55 | 3.65 ± 1.02 | 0.14 | 0.1144 |
| 2H-Pyran-2-one, tetrahydro-6-methyl- | 26.83 | creamy, fruity, coconut | 5.15 ± 2.55a | 3.14 ± 0.93bc | 2.88 ± 0.68c | 3.74 ± 0.36abc | 4.43 ± 0.81ab | 0.26 | 0.0325 |
| 1-Nonanol | 5.30 | fresh, clean, fatty, floral, rose, orange, dusty, wet, oily | 2.89 ± 0.64 | 3.35 ± 0.57 | 3.42 ± 0.54 | 3.05 ± 0.49 | 3.37 ± 1.01 | 0.12 | 0.6313 |
| 2-pentyl-Furan | 6.00 | fruity, green, earthy, beany, vegetable, metallic | 8.26 ± 1.83a | 3.95 ± 0.96b | 4.43 ± 1.08b | 4.31 ± 0.67b | 5.12 ± 1.44b | 0.35 | ＜0.0001 |
| Decanoic acid ethyl ester | 5.00 | sweet, waxy, fruity, apple, grape, oily, brandy | 3.75 ± 1.43 | 12.72 ± 15.84 | 18.91 ± 38.95 | 8.35 ± 14.74 | 7.49 ± 10.84 | 3.73 | 0.7818 |
| 2-hydroxy-Benzoic acid, pentyl ester | 7.50 | herbal, floral, clover, azalea, green, sweet, chocolate | 1.61 ± 1.06 | 2.07 ± 0.40 | 1.66 ± 0.52 | 1.84 ± 1.16 | 1.67 ± 0.99 | 0.15 | 0.8941 |
| 2-Undecenal | 10.00 | fresh, fruity, orange, peel | 3.80 ± 1.24 | 3.45 ± 0.39 | 3.00 ± 1.17 | 3.67 ± 0.87 | 3.68 ± 0.71 | 0.17 | 0.6012 |
| Tridecane | 42.00 | alkane | 2.24 ± 0.51b | 2.07 ± 0.61b | 1.84 ± 0.25b | 2.96 ± 0.49a | 2.99 ± 0.56a | 0.12 | 0.0009 |
| Citronellol | 40.00 | floral, rose, lime | 0.92 ± 0.58 | 2.56 ± 1.63 | 3.06 ± 0.91 | 1.63 ± 1.12 | 1.95 ± 1.34 | 0.25 | 0.0575 |
| Octanoic acid, ethyl ester | 40.00 | fruity, banana | 1.97 ± 0.40 | 2.51 ± 1.11 | 2.33 ± 2.04 | 2.78 ± 0.89 | 3.32 ± 0.58 | 0.22 | 0.4056 |
| 2-ethyl-Phenol | 30.00 | phenol | 2.13±0.56 | 2.06 ± 0.48 | 1.95 ± 0.24 | 2.21 ± 0.22 | 2.68 ± 0.53 | 0.09 | 0.0582 |
| 1-Decanol | 23.00 | fatty, waxy, floral, orange, sweet, watery | 3.02 ± 1.40 | 1.96 ± 0.45 | 1.99 ± 0.50 | 3.38 ± 2.24 | 2.58 ± 0.34 | 0.24 | 0.2254 |
| 2-methyl-3-Pentanol | 420.00 | - | 2.18 ± 0.11 | 2.22 ± 0.26 | 2.13 ± 0.22 | 2.25 ± 0.11 | 2.24 ± 0.14 | 0.03 | 0.7927 |
| n-butyl-Benzene | 14.00 | - | 1.64 ± 0.41b | 1.53 ± 0.33b | 1.52 ± 0.17b | 1.71 ± 0.22ab | 2.08 ± 0.40a | 0.07 | 0.0363 |
| 1,2,4,5-tetramethyl-Benzene | 87.00 | rancid, sweet | 1.21 ± 0.41 | 0.94 ± 0.21 | 0.65 ± 0.10 | 2.97 ± 3.42 | 2.06 ± 0.69 | 0.32 | 0.1185 |
| Naphthalene | 50.00 | pungent, dry, tarry | 1.55 ± 0.35a | 1.09 ± 0.30b | 0.72 ± 0.11b | 1.78 ± 0.53a | 1.63 ± 0.28a | 0.10 | 0.0001 |
| 1,3-bis(1,1-dimethylethyl)-Benzene | 1100.00 | - | 1.6 ± 0.31ab | 1.41 ± 0.21b | 1.31 ± 0.30b | 1.55 ± 0.15ab | 1.76 ± 0.17a | 0.05 | 0.0306 |
| Butanoic acid, 3-methyl-, butyl ester | 78.00 | apple, pear, fruity, sweet, pineapple, green, peach | 1.87 ± 0.30 | 1.37 ± 0.17 | 1.32 ± 0.31 | 1.93 ± 0.87 | 1.64 ± 0.07 | 0.09 | 0.0891 |
| 2(4H)-Benzofuranone, 5,6,7,7a-tetrahydro-4,4,7a-trimethyl-, (R)- | 2.10 | musky, coumarin | 1.75 ± 0.74 | 1.31 ± 0.43 | 1.24 ± 0.42 | 2.40 ± 1.72 | 1.55 ± 0.40 | 0.17 | 0.2118 |
| 3-Hexanol | 820.50 | alcoholic, fusel, rummy, whiskey, guava, apple | 1.17 ± 0.06 | 1.18 ± 0.14 | 1.10 ± 0.09 | 1.25 ± 0.07 | 1.23 ± 0.07 | 0.02 | 0.0649 |
| 2-Undecanol | 8.60 | fresh, waxy, clean, cloth, cotton, sarsaparilla | 1.09 ± 1.09 | 1.42 ± 0.43 | 1.07 ± 0.55 | 0.95 ± 0.17 | 1.20 ± 0.43 | 0.08 | 0.3554 |
| 4-Octanone | 41.00 | - | 1.59 ± 0.24a | 1.13 ± 0.11b | 1.06 ± 0.29b | 1.04 ± 0.40b | 1.13 ± 0.19b | 0.06 | 0.0140 |
| Isophorone | 11.00 | cool, woody, sweet, green, camphor, fruity, musty, cedarwood, tobacco, leathery | 3.28 ± 4.82 | 0.61 ± 0.42 | 0.89 ± 0.53 | 0.90 ± 0.57 | 1.54 ± 1.39 | 0.40 | 0.2688 |
| 2-Undecanone | 6.20 | waxy, fruity, creamy, fatty, orris, floral | 1.26 ± 0.54 | 1.15 ± 0.18 | 0.98 ± 0.27 | 1.26 ± 0.36 | 1.11 ± 0.16 | 0.06 | 0.5691 |
| 2-Nonanol | 58.00 | rose | 0.76 ± 0.26 | 0.69 ± 0.21 | 0.89 ± 0.34 | 0.81 ± 0.34 | 1.02 ± 0.27 | 0.05 | 0.3562 |

Table S6. List of 202 candidate key regulatory genes identified by intersecting flavor-associated WGCNA modules with differentially expressed genes.

| **Ensembl Gene ID** | **Gene Symbol** | **Ensembl Gene ID** | **Gene Symbol** | **Ensembl Gene ID** | **Gene Symbol** |
| --- | --- | --- | --- | --- | --- |
| ENSSSCG00000017693 | AATF | ENSSSCG00000025588 | FJX1 | ENSSSCG00000004473 | PHIP |
| ENSSSCG00000012006 | ABCC13 | ENSSSCG00000007079 | FLRT3 | ENSSSCG00000008468 | PKDCC |
| ENSSSCG00000001782 | ABHD17C | ENSSSCG00000015267 | FMO2 | ENSSSCG00000005657 | PKN3 |
| ENSSSCG00000031728 | ABRA | ENSSSCG00000017131 | FN3K | ENSSSCG00000007032 | PLAT |
| ENSSSCG00000037559 | ACOT11 | ENSSSCG00000011195 | GALNT15 | ENSSSCG00000022784 | PLEKHO1 |
| ENSSSCG00000006788 | ADORA3 | ENSSSCG00000023142 | GAMT | ENSSSCG00000005220 | PLPP6 |
| ENSSSCG00000005627 | AK1 | ENSSSCG00000014329 | GFRA3 | ENSSSCG00000011690 | PLSCR4 |
| ENSSSCG00000025578 | ALDH1A2 | ENSSSCG00000027196 | GIMAP6 | ENSSSCG00000036244 | PRR33 |
| ENSSSCG00000010404 | ALOX5 | ENSSSCG00000056302 | GINS2 | ENSSSCG00000005680 | PRRX2 |
| ENSSSCG00000030156 | APBB1IP | ENSSSCG00000036239 | GJA8 | ENSSSCG00000026554 | PRXL2B |
| ENSSSCG00000014156 | ARRDC3 | ENSSSCG00000023328 | GPM6A | ENSSSCG00000005252 | PTAR1 |
| ENSSSCG00000029474 | AVEN | ENSSSCG00000029592 | GPRC5A | ENSSSCG00000010755 | PTPRE |
| ENSSSCG00000039681 | B9D1 | ENSSSCG00000005164 | HACD4 | ENSSSCG00000000138 | PVALB |
| ENSSSCG00000000111 | BAIAP2L2 | ENSSSCG00000026733 | HIPK2 | ENSSSCG00000036352 | RAB7B |
| ENSSSCG00000001958 | BAZ1A | ENSSSCG00000028523 | HUS1 | ENSSSCG00000016824 | RAI14 |
| ENSSSCG00000029163 | BCAT1 | ENSSSCG00000058216 | IGFBP3 | ENSSSCG00000021591 | RBM3 |
| ENSSSCG00000009629 | BIN3 | ENSSSCG00000028331 | IL1R2 | ENSSSCG00000054936 | RBP5 |
| ENSSSCG00000009620 | BMP1 | ENSSSCG00000034720 | IQCK | ENSSSCG00000006335 | RGS4 |
| ENSSSCG00000007501 | BMP7 | ENSSSCG00000014088 | IQGAP2 | ENSSSCG00000016395 | RIF1 |
| ENSSSCG00000001422 | C2 | ENSSSCG00000000475 | IRAK3 | ENSSSCG00000005858 | RNF208 |
| ENSSSCG00000001427 | C4A | ENSSSCG00000016009 | ITGA4 | ENSSSCG00000026427 | RORC |
| ENSSSCG00000004565 | CA12 | ENSSSCG00000022209 | ITGB1BP2 | ENSSSCG00000006582 | S100A14 |
| ENSSSCG00000001100 | CARMIL1 | ENSSSCG00000004093 | IYD | ENSSSCG00000021651 | SCN2B |
| ENSSSCG00000059202 | CCDC71L | ENSSSCG00000031899 | JSRP1 | ENSSSCG00000016969 | SERF1A |
| ENSSSCG00000013593 | CCL25 | ENSSSCG00000035667 | KBTBD13 | ENSSSCG00000011740 | SERPINI1 |
| ENSSSCG00000011322 | CCR1 | ENSSSCG00000026680 | KCND3 | ENSSSCG00000012160 | SH3KBP1 |
| ENSSSCG00000013579 | CD209 | ENSSSCG00000025901 | LCLAT1 | ENSSSCG00000005949 | SLA |
| ENSSSCG00000039640 | CDC34 | ENSSSCG00000028512 | LDLR | ENSSSCG00000059879 | SLC2A1 |
| ENSSSCG00000040183 | CDK6 | ENSSSCG00000025188 | LEPR | ENSSSCG00000008550 | SLC5A6 |
| ENSSSCG00000003459 | CELA2A | ENSSSCG00000003157 | LIN7B | ENSSSCG00000011600 | SLC6A6 |
| ENSSSCG00000003403 | CENPS | ENSSSCG00000023391 | LONP2 | ENSSSCG00000029456 | SLC7A1 |
| ENSSSCG00000059665 | CEP192 | ENSSSCG00000008701 | LRPAP1 | ENSSSCG00000011641 | SLCO2A1 |
| ENSSSCG00000033574 | CFAP54 | ENSSSCG00000016589 | LRRC4 | ENSSSCG00000012046 | SMIM11 |
| ENSSSCG00000017098 | CFAP90 | ENSSSCG00000011401 | LSMEM2 | ENSSSCG00000004233 | SMPDL3A |
| ENSSSCG00000032532 | CHRM2 | ENSSSCG00000007853 | LYRM1 | ENSSSCG00000005471 | SNX30 |
| ENSSSCG00000039677 | CHRNA4 | ENSSSCG00000013043 | MACROD1 | ENSSSCG00000000867 | SPIC |
| ENSSSCG00000036132 | CKM | ENSSSCG00000003143 | MAMSTR | ENSSSCG00000006277 | SPIDR |
| ENSSSCG00000058408 | COA5 | ENSSSCG00000030354 | MAP3K2 | ENSSSCG00000032423 | SPOCK1 |
| ENSSSCG00000004484 | COL12A1 | ENSSSCG00000020705 | MAP3K8 | ENSSSCG00000009216 | SPP1 |
| ENSSSCG00000023630 | CPM | ENSSSCG00000002962 | MAP4K1 | ENSSSCG00000009642 | STC1 |
| ENSSSCG00000008187 | CRACDL | ENSSSCG00000004939 | MEGF11 | ENSSSCG00000029304 | STEAP3 |
| ENSSSCG00000001770 | CTSH | ENSSSCG00000034639 | MGAM | ENSSSCG00000005376 | TBC1D2 |
| ENSSSCG00000022009 | DDC | ENSSSCG00000000368 | MMP19 | ENSSSCG00000014894 | TENM4 |
| ENSSSCG00000012850 | DEAF1 | ENSSSCG00000040160 | MMP24 | ENSSSCG00000009157 | TET2 |
| ENSSSCG00000009431 | DGKH | ENSSSCG00000004620 | MYO5A | ENSSSCG00000038138 | TFEC |
| ENSSSCG00000004441 | DSE | ENSSSCG00000015559 | NCF2 | ENSSSCG00000007671 | TFR2 |
| ENSSSCG00000034147 | DTYMK | ENSSSCG00000039311 | NCS1 | ENSSSCG00000026043 | TGM3 |
| ENSSSCG00000040815 | DUSP5 | ENSSSCG00000038614 | NENF | ENSSSCG00000017810 | TLCD3A |
| ENSSSCG00000031421 | DYNLRB2 | ENSSSCG00000006474 | NES | ENSSSCG00000005503 | TLR4 |
| ENSSSCG00000036520 | EFNA5 | ENSSSCG00000005022 | NIN | ENSSSCG00000000927 | TMTC3 |
| ENSSSCG00000029484 | EMB | ENSSSCG00000031142 | NMNAT3 | ENSSSCG00000034615 | TNFAIP8L1 |
| ENSSSCG00000026812 | EMG1 | ENSSSCG00000040860 | NMRK2 | ENSSSCG00000011358 | TREX1 |
| ENSSSCG00000009975 | EMID1 | ENSSSCG00000010990 | NOL6 | ENSSSCG00000039862 | TRIB3 |
| ENSSSCG00000017904 | ENO3 | ENSSSCG00000023527 | NRROS | ENSSSCG00000031531 | TSPAN10 |
| ENSSSCG00000025992 | ENPP3 | ENSSSCG00000028210 | NT5C | ENSSSCG00000003875 | TTC39A |
| ENSSSCG00000026167 | - | ENSSSCG00000000896 | NTN4 | ENSSSCG00000016722 | UBE2D4 |
| ENSSSCG00000028674 | - | ENSSSCG00000046640 | OAS1 | ENSSSCG00000016510 | UBN2 |
| ENSSSCG00000038719 | - | ENSSSCG00000008829 | OCIAD2 | ENSSSCG00000014833 | UCP2 |
| ENSSSCG00000040448 | - | ENSSSCG00000034510 | ORAI1 | ENSSSCG00000030388 | UPP1 |
| ENSSSCG00000042192 | - | ENSSSCG00000016851 | OSMR | ENSSSCG00000011120 | USP6NL |
| ENSSSCG00000043595 | - | ENSSSCG00000024072 | P2RY11 | ENSSSCG00000002696 | VAT1L |
| ENSSSCG00000054645 | - | ENSSSCG00000040207 | P2RY2 | ENSSSCG00000023837 | WFDC1 |
| ENSSSCG00000057192 | - | ENSSSCG00000040507 | PDLIM7 | ENSSSCG00000032452 | WFS1 |
| ENSSSCG00000057525 | - | ENSSSCG00000001869 | PEAK1 | ENSSSCG00000035524 | WNT9B |
| ENSSSCG00000058502 | - | ENSSSCG00000009631 | PEBP4 | ENSSSCG00000037815 | ZC3H12A |
| ENSSSCG00000062952 | - | ENSSSCG00000016720 | PGAM2 | ENSSSCG00000022989 | ZNF704 |
| ENSSSCG00000029598 | FAM124A | ENSSSCG00000008773 | PGM2 |  |  |
| ENSSSCG00000016865 | FBXO4 | ENSSSCG00000030467 | PHC3 |  |  |

**Fig S1**. Statistical comparison of E-nose sensor responses. Data are mean ± SEM (*n* = 5/6). Different letters denote significant differences (*P* < 0.05) among groups.

**Fig S2**. Size distribution of the metabolite co-abundance modules identified by WGCNA.

**Fig S3**. Functional enrichment analysis and gene co-expression module characterization. (**A**) GSEA of the transcriptome (MS vs. LR) using the Reactome database. (**B**) GSEA of the transcriptome (MS vs. LR) using the Wikipathways database. (**C**) Size distribution of gene co‑expression modules identified by transcriptomic WGCNA.

**Fig S4**. Functional characterization and sequence analysis of *ALOX5* in relation to flavor formation. (**A**) Correlation between *ALOX5* expression and the four characteristic flavor compounds. Scatter plots show the linear relationship between muscle *ALOX5* mRNA level (TPM) and the abundance of each key flavor compound across all 29 pigs, with Pearson's r and *P*-value indicated.. (**B**) Sequence alignment of the ALOX5 protein between pig and mouse. (**C**) *ALOX5* expression at both the transcriptional and translational levels in C2C12 cells treated with oleic or α‑linolenic acid. (**D**) Cell viability assay for fatty acid treatments.
